# Immune Infiltration Characteristics and a Gene Prognostic Signature Associated With the Immune Infiltration in Head and Neck Squamous Cell Carcinoma

**DOI:** 10.3389/fgene.2022.848841

**Published:** 2022-05-02

**Authors:** Chunmei Zhu, Qiuji Wu, Ningning Yang, Zhewen Zheng, Fuxiang Zhou, Yunfeng Zhou

**Affiliations:** ^1^ Department of Radiation and Medical Oncology, Zhongnan Hospital of Wuhan University, Wuhan, China; ^2^ Hubei Key Laboratory of Tumor Biological Behaviors, Zhongnan Hospital of Wuhan University, Wuhan, China; ^3^ Hubei Cancer Clinical Study Center, Zhongnan Hospital of Wuhan University, Wuhan, China

**Keywords:** head and neck squamous cell carcinoma, immune cell infiltration, prognosis, response to immunotherapy, immune inhibitor receptor

## Abstract

**Background:** Immunotherapy has become the new standard of care for recurrent and metastatic head and neck squamous cell carcinoma (HNSCC), and PD-L1 is a widely used biomarker for immunotherapeutic response. However, PD-L1 expression in most cancer patients is low, and alternative biomarkers used to screen the population benefiting from immunotherapy are still being explored. Tumor microenvironment (TME), especially tumor immune-infiltrating cells, regulates the body’s immunity, affects the tumor growth, and is expected to be a promising biomarker for immunotherapy.

**Purpose:** This article mainly discussed how the immune-infiltrating cell patterns impacted immunity, thereby affecting HNSCC patients’ prognosis.

**Method:** The immune-infiltrating cell profile was generated by the CIBERSORT algorithm based on the transcriptomic data of HNSCC. Consensus clustering was used to divide groups with different immune cell infiltration patterns. Differentially expressed genes (DEGs) obtained from the high and low immune cell infiltration (ICI) groups were subjected to Kaplan–Meier and univariate Cox analysis. Significant prognosis-related DEGs were involved in the construction of a prognostic signature using multivariate Cox analysis.

**Results:** In our study, 408 DEGs were obtained from high- and low-ICI groups, and 59 of them were significantly associated with overall survival (OS). Stepwise multivariate Cox analysis developed a 16-gene prognostic signature, which could distinguish favorable and poor prognosis of HNSCC patients. An ROC curve and nomogram verified the sensitivity and accuracy of the prognostic signature. The AUC values for 1 year, 2 years, and 3 years were 0.712, 0.703, and 0.700, respectively. TCGA-HNSCC cohort, GSE65858 cohort, and an independent GSE41613 cohort proved a similar prognostic significance. Notably, the prognostic signature distinguished the expression of promising immune inhibitory receptors (IRs) well and could predict the response to immunotherapy.

**Conclusion:** We established a tumor immune cell infiltration (TICI)-based 16-gene signature, which could distinguish patients with different prognosis and help predict the response to immunotherapy.

## Introduction

Head and neck squamous cell carcinoma (HNSCC) is categorized into oral cavity, nasal cavity, nasopharynx, oropharynx, hypopharynx, larynx, and others. It ranks as the 10th most common malignancy with 600,000 new cases worldwide reported each year (Ferlay et al., 2015). Smoking and drinking are considered to be the two main causes of HNSCC. Recently, accumulating evidence has established a causal role of high-risk human papillomavirus (HPV) infections in the etiology of HNSCC (Castellsagué et al., 2016). HPV-related HNSCC displays significantly increased sensitivity to chemoradiotherapy and is associated with improved prognosis (Chaturvedi et al., 2011; Dok et al., 2020).

Conventional therapies such as surgery and radiotherapy form the basis of early-stage HNSCC treatment, with the 5-year OS reaching 80–90% for surgery and 65–80% for radiotherapy. Despite the extended screening and improvement in treatment in the past few years, more than 50% of HNSCC patients are at an advanced stage when diagnosed, and the 5-year survival rate is only 34.9% (Chauhan et al., 2015). More than 50% of locally advanced patients would develop recurrence or distant metastasis within 2 years, following radical treatment (Sacco and Cohen, 2015). For patients with the late stage, the treatment opportunity is limited after first-line treatment failure. The prognosis of these patients is extremely poor. The 5-year survival rate of these patients is only 3.6%, and the median survival time is less than half a year (Machiels et al., 2015).

In recent years, immunotherapy has achieved great success in a variety of tumors such as non–small cell lung cancer (NSCLC), triple-negative breast cancer (TNBC), melanoma, and other tumors (Luke et al., 2017; Migden et al., 2018; West et al., 2019; Mansfield et al., 2020; Schmid et al., 2020; Paz-Ares et al., 2021). The use of immune checkpoint inhibitors both as second-line and first-line treatments has also led to significant improvement in HNSCC patient prognosis (Ferris et al., 2016; Bauml et al., 2017; Burtness et al., 2019). However, only a part of HNSCC patients could benefit from immunotherapy; most patients have primary resistance or gradual resistance to immunotherapy. The exact mechanism remains incompletely illustrated. Recent studies suggested that the response to immunotherapy might rely on tumor microenvironment (TME). TME was composed of complex components such as extracellular matrix, stromal cells, endothelial cells, immune cells, and various soluble molecules (Wang et al., 2020). Among them, immune-infiltrating cells, including T cells, NK cells, B cells, and macrophages, were the most active and acted directly on tumors. The heterogeneity of tumor immune cell infiltration was another vital factor that determined the best response to the patient’s immunotherapy. Patients with enriched T-cell infiltration might respond better to immunotherapy. On the contrary, patients with poor T-cell infiltration might be resistant to immunotherapy, and additional intervention was needed for those patients (Gajewski et al., 2013; Srinivasan et al., 2018).

Moreover, immune-infiltrating cells proved to be an independent prognostic factor in cancers. For instance, increased infiltration of NK cells was correlated with an improved survival of melanoma, hematological malignancies, and other solid tumors (Fang et al., 2017). Tumor-infiltrating CD4^+^T cells and CD8^+^T cells were correlated with superior prognosis of breast cancer, colorectal cancer, glioblastoma, and cervical cancers (Saito et al., 2016; Maimela et al., 2019).

The TME analysis of HNSCC also proved that immune-infiltrating cells, cytokines, and immunomodulatory molecules determine the host’s antitumor immune response ability (Ferris, 2015; Chen and Mellman, 2017). Therefore, a better understanding of the TME, especially the tumor-infiltrating cells, is essential for improving response to immunotherapy and the prognosis of HNSCC patients. In this study, we used the CIBERSORT algorithm to generate a tumor immune cell infiltration profile of HNSCC and then explored the potential relationship between immune-infiltrating cells and patients’ prognosis and immunotherapeutic response. We hope this study will provide valuable insights into the complex tumor immune microenvironment and help us understand how immune status affects cancer cells and immunotherapy.

## Materials and Methods

### Data Acquisition and Processing

**FIGURE 1:**
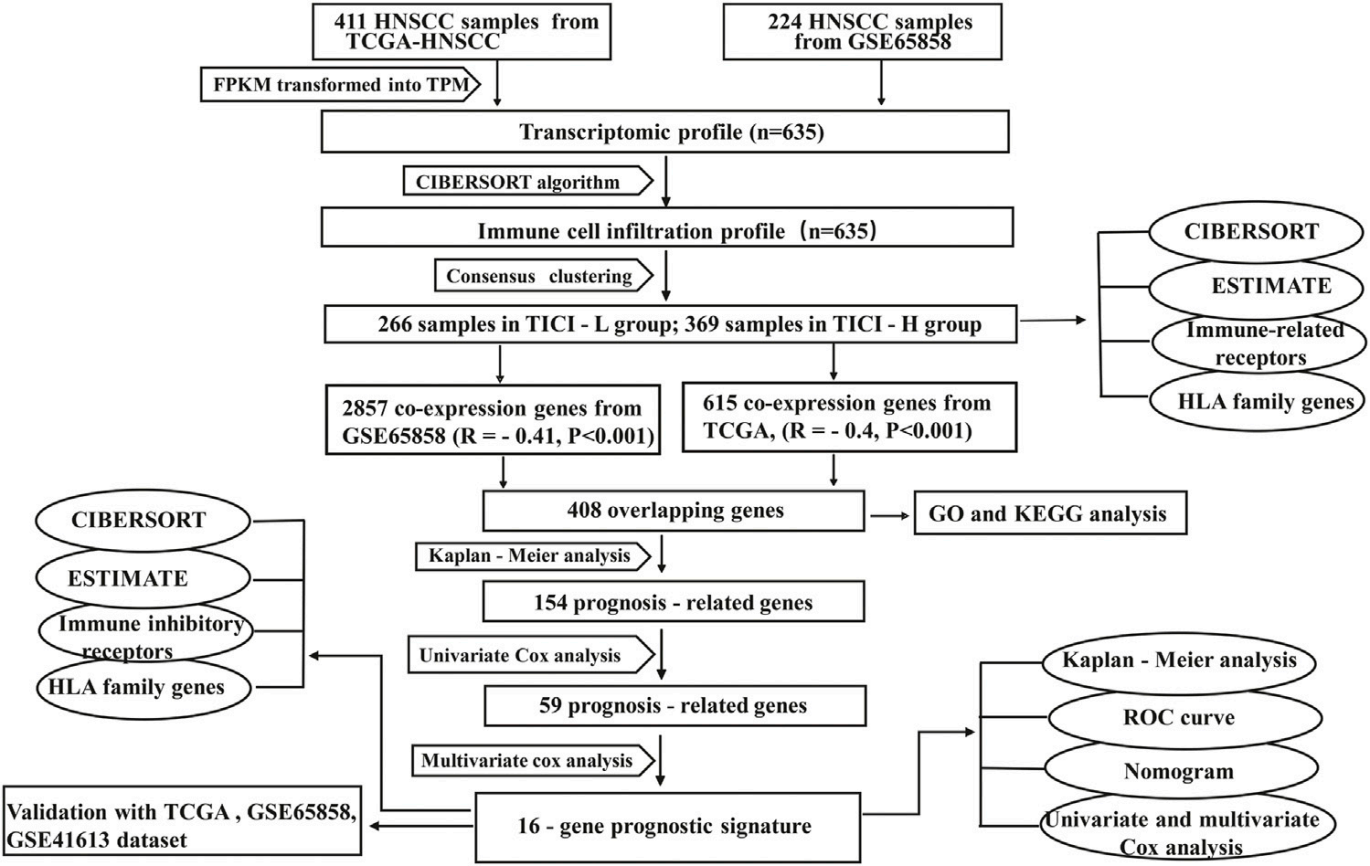
Workflow for our study. TICI, tumor immune cell infiltration.

The HNSCC RNA-seq data and the corresponding clinical information were downloaded from The Cancer Genome Atlas database (TCGA, https://portal.gdc.cancer.gov/) and Gene Expression Omnibus database (GEO, https://www.ncbi.nlm.nih.gov/gds). The GSE65858 dataset generated by Illumina was processed using the lumi software package. The GSE41613 dataset from Affymetrix was processed using the RMA algorithm. As for TCGA-HNSCC microarray data, RNA-seq data in the form of fragments per kilobase of transcript per million mapped reads (FPKM) were adjusted to the form of transcripts per kilobase of transcript per million mapped reads (TPM) using the function tpm in the edge package. We presented the expression profile of TCGA cohort similar with the results from the GSE65858 cohort and GSE41613 cohort, so that the data were comparable among samples (Wagner et al., 2012; Zeng et al., 2019). The batch effects between different datasets within the same platform were adjusted by the ComBat method (Leek et al., 2012). The workflow of our study was shown in Figure 1.

### Consensus Clustering for Immune-Infiltrating Cells

The CIBERSORT algorithm (Newman et al., 2015) was applied to generate the immune cell infiltration profiles. Then, consensus clustering was performed to determine different immune cell infiltration patterns using the ConsensusClusterPlus package (Wilkerson and Hayes, 2010). The ESTIMATE algorithm (Yoshihara et al., 2013) was used to generate immune score, estimate score, and stromal score using the “ESTIMATE” package of R.

### Weighted Gene Co-expression Network Analysis

The weighted gene co-expression network analysis (WGCNA) was used to identify the co-expressed gene modules using the WGCNA package of R. A threshold was set to β = 0.5 to ensure a scale-free network. The dendrogram visually displayed the clustering of genes, and the heatmaps showed the correlation between co-expressed gene modules and TICI. In our study, positive correlation modules with high relevant coefficients from the GSE65858 cohort and TCGA cohort were identified. Finally, the overlapping co-expressed genes from these two cohorts were visualized as Venn diagram using the VennDiagram package of R and were used for stepwise prognosis-related gene identification.

### Functional and Pathway Annotation

The gene ontology (GO) function and the Kyoto Encyclopedia of Genes and Genomes (KEGG) pathway enrichment analysis were performed for the overlapping co-expressed genes using the clusterProfiler package.

### Establishment of the Prognostic Signature and Preliminary Exploration of the Genes of the Signature

Kaplan–Meier and univariate Cox regression analyses were performed to identify the prognosis-related genes. With multivariate Cox analysis, we developed a prognostic signature. The risk formula is as follows:

$$Risk\;score=\overset{n}{\underset{i=1}{\sum }}coef_{i}\text{∗}exp_{i}$$

Coef_i_, exp_i_, and n represented the multivariate Cox regression coefficient, the gene expression value, and the number of genes in the prognostic signature, respectively. Next, the roles of the genes in the prognostic signature were explored in mRNA and protein expression levels with the HPA database (https://www.proteinatlas.org/) (Uhlen et al., 2010).

### Verification of the Prognostic Signature

The risk scores of the 635 HNSCC patients were calculated according to the risk formula, and the HNSCC patients were assigned into high- and low-risk groups according to the optimal cutoff value of the risk score based on the maximum value of (sensitivity + specificity-1) in the ROC curve (Youden index) using the surv_cutpoint function of the survminer R package. Kaplan–Meier analysis, risk curves, ROC curves, nomogram, and univariate and multivariate Cox analyses were used to evaluate the prognostic signature. In addition, TCGA-HNSCC subgroup, GSE65858 subgroup, and an external GSE41613 cohort were used as the validation groups to verify the gene prognostic signature.

Since the prognostic signature was developed based on the TICI, the correlation analysis of signature genes with immune-infiltrating cells and immune score was performed. The expression of immune inhibitor receptors (PD-L1, PD-1, CTLA-4, LAG3, HAVCR2, and TIGIT) in the high- and low-risk groups was also compared. Moreover, the Tumor IMmune Estimation Resource database (TIMER: https://cistrome.shinyapps.io/timer/) (Li et al., 2017) was used to investigate the association between prognostic genes of the signature and immune-infiltrating cells (CD8^+^T cells, CD4^+^T cells, B cells, macrophages, neutrophils, and dendritic cells).

### Mutation and Prognostic Signature

The Masked Somatic Mutation data (VarScan) of HNSCC was downloaded from TCGA database and was processed using the maftools package of R (Mayakonda et al., 2018). The mutation characteristics in HNSCC were analyzed, and the correlation analysis between mutation and risk score was performed.

### Gene Set Enrichment Analysis

GSEA (version GSEA 4.1.0) was performed to annotate the function and pathway enrichment of the prognostic signature.

### Statistical Analysis

Perl was used for data processing. R (MathSoft, version 4.0.3) was used for plotting and statistical analysis. The packages used were as follows: limma, pheatmap, ggplot2, org.Hs.eg.db, clusterProfiler, VennDiagram, WGCNA, preprocessCore, estimate, enrichplot, survival, glmnet, survminer, survivalROC, beeswarm, and rms. Meta-analysis was performed to assess heterogeneity of different cohorts and to generate the hazard rate of the risk score of each dataset using the Stata software (Texas, U.S., StataIC 15).

The Mann–Whitney test was performed for continuous variables of two groups, and the Kruskal–Wallis test was performed for continuous variables of multiple groups (with the Bonferroni correction for pairwise comparisons among multiple groups). The log-rank test and Cox regression were used for survival analysis. All tests were two-sided, and for all statistical tests, a value of *p* < 0.05 was considered statistically significant unless otherwise specified.

## Results

### Establishment and Evaluation of TICI-Related Groups

**FIGURE 2:**
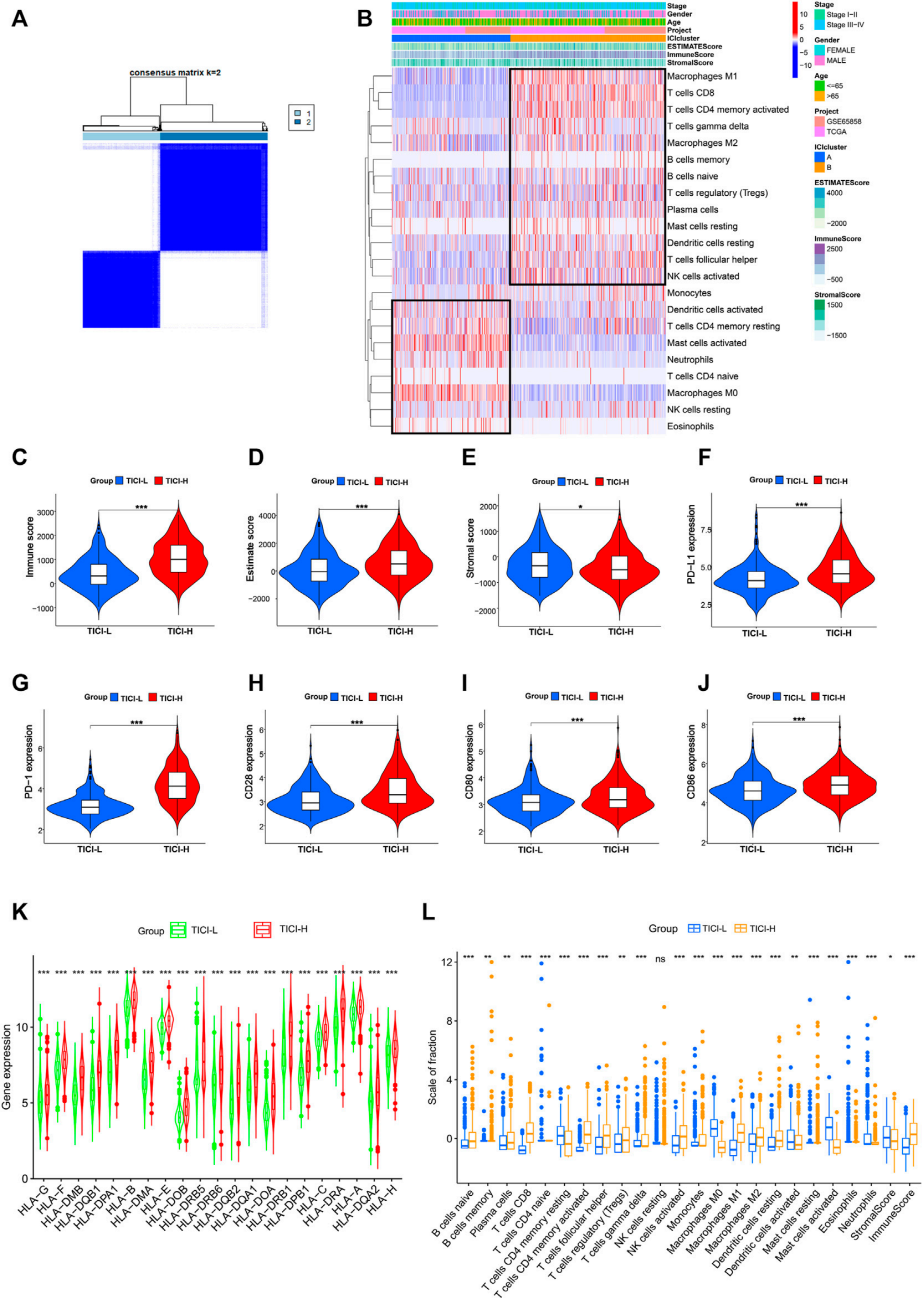
**(A)** Consensus clustering matrix for *k* = 2. **(B)** Heatmap of immune-infiltrating cells. **(C–E)** Immune score, estimate score, and stromal score in the high- and low-TICI groups. **(F–J)** Expression of PD-L1, PD-1, CD28, CD80, and CD86 in the high- and low-TICI groups. **(K)** Expression of HLA genes in the high- and low-TICI groups. **(L)** Immune-infiltrating cells in the high- and low-TICI groups.

The immune cell infiltration profile was generated based on the transcriptomic data of TCGA cohort (*n* = 411) and GSE65858 cohort (*n* = 224). The clinical characteristics of these two cohorts are shown in Table 1. The patients were divided into two immune infiltration patterns using consensus clustering (Figure 2A). In our study, the cluster with high infiltration of CD8^+^T cells, activated memory CD4^+^T cells, activated NK cells, follicular helper T cells, memory B cells, naive B cells, plasma cells, and M1 and M2 macrophages was named the high tumor immune cell infiltration (TICI) group. On the other hand, the cluster with low infiltration of the aforementioned immune cells but with high infiltration of resting immune cells or inflammatory cells, such as resting memory CD4^+^T, resting NK cells, neutrophils, M0 macrophages, and mast cells, was named the low TICI group (Figure 2B). Kaplan–Meier analysis of these tumor immune-infiltrating cells indicated that high infiltration of activated CD4^+^T cells, CD8^+^T cells, follicular helper T cells, and naive B cells was related with favorable prognosis, while high infiltration of M0 macrophages, neutrophils, and mast cells was related with poor prognosis (Supplementary Figure S1). Perhaps not surprised, the high-ICI group was associated with an improved survival rate, and the low-TICI group was related with a poor survival rate (Supplementary Figure S2).

**TABLE 1:** Characteristics of the 635 HNSCC patients.

Characteristic	Clinical feature	Total	GSE65858	TCGA-HNSCC	*p*-value
Gender	Female	151(23.78%)	38(16.96%)	113(27.49%)	0.004
	Male	484(76.22%)	186(83.04%)	298(72.51%)	
Age	≤65	408(64.25%)	148(66.07%)	260(63.26%)	0.536
	>65	227(35.75%)	76(33.93%)	151(36.74%)	
Stage^a^	Stage I–II	128(20.16%)	45(20.09%)	83(20.19%)	0.399
	Stage III–IV	450(70.87%)	179(79.91%)	271(65.94%)	
	Unknown	57(8.98%)	0(0%)	57(13.87%)	
T stage^a^	T1-2	255(40.16%)	98(43.75%)	157(38.2%)	0.951
	T3-4	333(52.44%)	126(56.25%)	207(50.36%)	
	Unknown	47(7.4%)	0(0%)	47(11.44%)	
N stage^a^	N0	203(31.97%)	72(32.14%)	131(31.87%)	0.080
	N1-3	350(55.12%)	152(67.86%)	198(48.18%)	
	Unknown	82(12.91%)	0(0%)	82(19.95%)	
M stage^a^	M0	368(57.95%)	218(97.32%)	150(36.5%)	0.109
	M1	6(0.94%)	6(2.68%)	0(0%)	
	Unknown	261(41.1%)	0(0%)	261(63.5%)	

a Staging according to the seventh edition AJCC guidelines.

The clinical characteristic differences between TCGA and GSE65858 datasets based on the χ2 test and with continuity correction where appropriate.

Next, we explored the immune features of these two ICI-related groups. First, the estimate score and immune score were higher in the high-ICI group, while the stromal score was higher in the low-ICI group (Figures 2C–E). The expression of immune-related genes such as PD-L1, PD-1, CD80, CD28, CD86, and human leukocyte antigen (HLA) family genes was also significantly higher in the high-ICI group (Figures 2F–K). The infiltration of the immune cells between the two groups is shown in Figure 2L.

Taken together, high- and low-ICI groups were presented with distinct immune cell infiltration characteristics, different immune-related molecule expression, and were associated with different prognosis. Compared with the low-TICI group, the high-TICI group might have good immunoreactivity.

### Weighted Gene Co-expression Network Analysis

**FIGURE 3:**
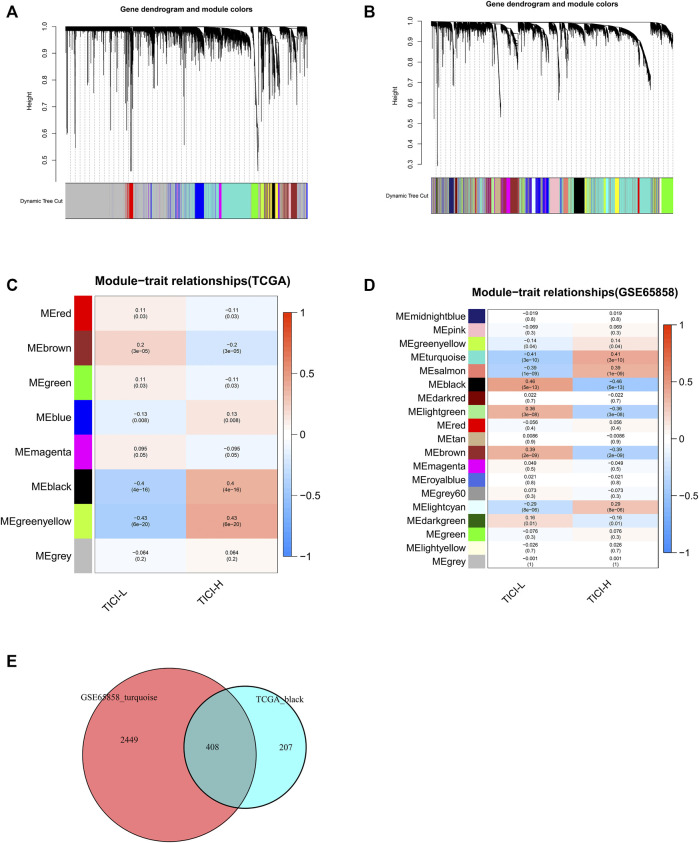
Identification of TICI-related co-expressed genes. **(A,B)** Clustering dendrograms of TCGA dataset **(A)** and GSE65858 dataset **(B)**. **(C,D)** Correlation heatmaps of different modules and TICI in TCGA dataset **(C)** and GSE65858 dataset **(D)**. **(E)** Identification of the overlapping co-expressed genes from the black module in TCGA dataset and the turquoise module in the GSE65858 dataset using VennDiagram software.

In TCGA dataset, the black module (|r| = 0.4, p = 4e^−16^) and green–yellow module (|r| = 0.43, p = 6e^−20^) were highly positively related with high-TICI compared with the other modules and were chosen as the candidate modules because the black module had much more TICI-related genes than the green–yellow module and was finally chosen for subsequent analysis. Similarly, the turquoise module (|r| = 0.41, p = 3e^−10^) in the GSE65858 dataset highly relevant to high-TICI was selected for subsequent analysis (Figures 3A–D). Ultimately, a total of 408 overlapping genes from the black module in TCGA dataset and the turquoise module in the GSE65858 dataset were obtained and participated in subsequent identification of prognosis-related genes (Figure 3E).

### GO and KEGG Analysis

**FIGURE 4:**
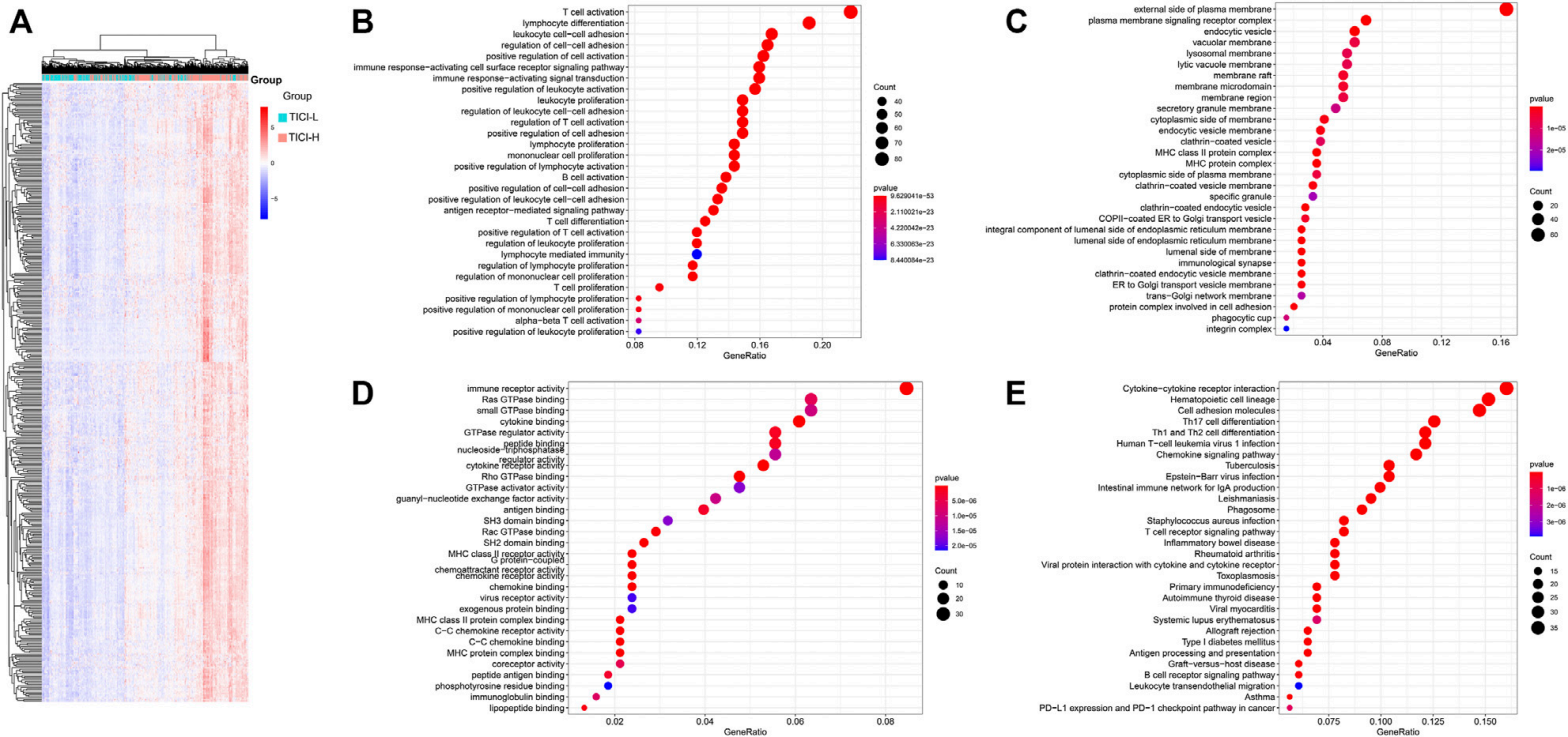
**(A)** Heatmap of the overlapping co-expressed genes; **(B–E)** GO and KEGG analysis for the overlapping co-expressed genes. BP **(B)**, CC **(C)**, MF **(D)**, and KEGG pathway **(E)**.

The expression of the 408 overlapping genes between high- and low-TICI groups is shown in Figure 4A. GO and KEGG analysis were performed to explore the biological characteristics of the 408 genes. For biological processes (BP), these genes mainly participated in T-cell activation, lymphocyte differentiation, leukocyte cell–cell adhesion, and lymphocyte and leukocyte proliferation. In terms of cellular components (CC), these genes were related to the MHC protein complex. The changes in molecular function (MF) showed that these genes were correlated with immune receptor activity, cytokine binding, MHC class II receptor activity, cytokine receptor activity, and MHC class II protein complex binding. The KEGG pathway analysis indicated that these genes were mainly enriched in signal pathways such as hematopoietic cell lineage, intestinal immune network for IgA production, cell adhesion molecules, and Th1, Th2, and Th17 cell differentiation (Figures 4B–E).

### Evaluation and Validation of the 16-Gene Prognostic Signature

Using multivariate Cox analysis, we obtained the following risk formula:

Risk score = (*P2RY8* ∗ 0.490331935) + (*FLT3LG* ∗ −0.363760832) + (*GIMAP1* ∗ 0.387873078) + (*CD79A* ∗ −0.168052271) + (*SLAMF6* ∗ 0.368179613) + (*FGD3* ∗ −0.238932879) + (*IKZF3* ∗ 0.193711011) + (*FAM107A* ∗ −0.263580347) + (*MAP4K1* ∗ 0.373764051) + (*GZMM* ∗ −0.223385774) + (*CCR7* ∗ −0.508653299) + (*P2RY10* ∗ 0.321200005) + (*XCR1* ∗ −0.277393854) + (*NLRC3* ∗ −0.374350099) + (*UBASH3A* ∗ −0.454714574) + (*ABCB1* ∗ −0.45779747).

**FIGURE 5:**
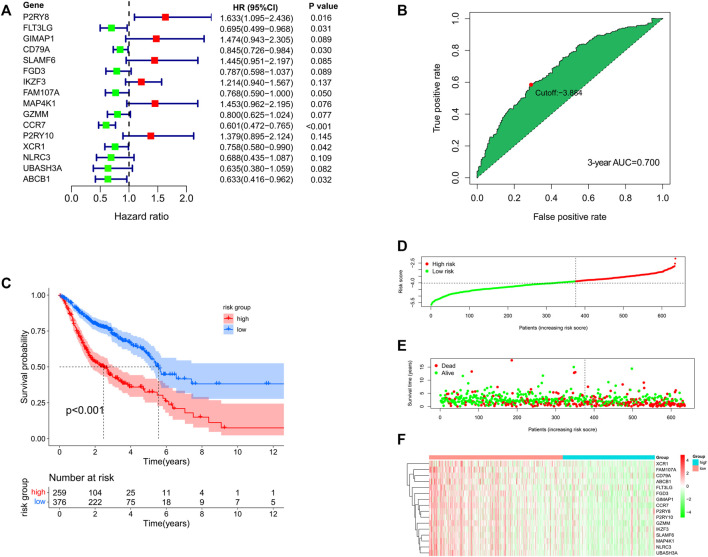
**(A)** Multivariate Cox analysis for the 16 genes in the prognostic signature. **(B)** Best cutoff value based on the ROC curve. **(C)** Kaplan–Meier analysis for the high- and low-risk groups. **(D–F)** Distribution of risk score, survival status, and the expression feature of the 16 prognostic genes of the 635 HNSCC patients.

Also, the multivariate Cox analysis of the 16 genes is shown in Figure 5A. Based on the risk formula, we calculated the risk score for each of the 635 HNSCC patients. According to the maximum value of the Youden index, we obtained the optimal cutoff value of the risk score, and the patients were divided into high- (*n* = 259) or low-risk group (*n* = 376) based on the cutoff value (Figure 5B). The Kaplan–Meier analysis showed that the low-risk group had significantly favorable prognosis compared with the high-risk group (Figure 5C). Moreover, the patients in the low-risk group also had better OS in different clinical subgroups (age (≤65 versus >65 years old), sex (male versus female), T stage (T1-2 versus T3-4), N stage (N0 versus N1-3), and pathological stage (stage I–II versus stage III–IV)) (Supplementary Figure S3). The AUC values were 0.712 for 1 year, 0.703 for 2 years, and 0.700 for 3 years (Supplementary Figure S4A). The risk score, the survival status, and the expression features of the 16 prognostic genes of the 635 HNSCC patients are shown in Figures 5D–F. In addition, we integrated age, gender, T stage, N stage, and risk score for prognosis analysis and drew an OS nomogram (Supplementary Figure S4B). The calibration curves further confirmed the prognostic value of the gene signature (Supplementary Figures S4C–E). Univariate Cox analysis indicated that the risk score (HR = 2.781, 95%CI: 2.132–3.626, *p* < 0.001) was closely related with the OS (Supplementary Figure S4F). When the clinical parameters (age, gender, pathology stage, T stage, and N stage) were included into the multivariate Cox regression analysis, we observed that the risk score (HR = 2.579, 95%CI = 1.953-3.404, *p* < 0.001) was an independent prognostic predictor (Supplementary Figure S4G).

In our study, TCGA subgroup, GSE65858 subgroup, and an independent GSE41613 dataset were used to further verify the prognostic signature. Perhaps not surprising, the OS of HNSCC patients in the low-risk group of TCGA cohort and GSE65858 cohort were significantly longer than that of the high-risk group (Supplementary Figures S5A,B). The 1-, 2-, and 3-year AUC values in TCGA cohort were 0.702, 0.701, and 0.703, respectively, and those in GSE65858 cohort were 0.737, 0.713, and 0.684, respectively (Supplementary Figures S5D,E). GSE41613 was an independent cohort composed of 97 HNSCC patients. According to the optimal cutoff value of the risk score, the patients in the GSE41613 cohort were assigned into a high- or low-risk group. Patients in the low-risk group had improved OS compared with the high-risk group (Supplementary Figure S5C). The 1-, 2- and 3-year AUC values of the GSE41613 cohort were 0.701, 0.600, and 0.567, respectively (Supplementary Figure S5F).

In order to confirm the predictive performance of these cohorts, meta-analysis was performed using the Stata software. Comparing the standardized mean difference (SMD) of these cohorts, we observed no obvious heterogeneity among TCGA cohort, GSE65858 cohort, the merger cohort (TCGA + GSE65858), or even GSE41613 cohort (*p* = 0.727) which indicated that these cohorts were comparable and the merger of TCGA cohort and GSE65858 cohort was reasonable (Supplementary Figure S5G). Considering that 1-, 2- and 3-year AUC values of the GSE41613 cohort were 0.701, 0.600, and 0.567, respectively, we performed multivariate Cox analysis on the HNSCC patients within 3-year OS. The result showed that the risk score was an independent risk factor with HR being 2.25 (95%CI: 1.81–3.34, *p* < 0.05) in TCGA cohort, HR being 3.12 (95%CI: 1.98–4.91, *p* < 0.05) in the GSE65858 cohort, HR being 2.63 (95%CI: 2.04–3.39, *p* < 0.05) in the merger cohort, HR being 1.80 (95%CI: 1.14–2.84, *p* < 0.05) in the GSE41613 cohort, and HR being 2.50 (95%CI: 2.11–2.97, *p* < 0.05) of the total combined effect (Supplementary Figure S5H).

### Preliminary Exploration of the Prognostic Genes in the Prognostic Signature

Next, the 408 overlapping genes were subjected into Kaplan–Meier analysis and univariate Cox analysis. A total of 59 prognosis-related genes were further identified (Table 2) and were introduced into multivariate Cox analysis. Finally, 16 prognosis-related genes (*P2RY8*, *FLT3LG*, *GIMAP1*, *CD79A*, *SLAMF6*, *FGD3*, *IKZF3*, *FAM107A*, *MAP4K1*, *GZMM*, *CCR7*, *P2RY10*, *XCR1*, *NLRC3*, *UBASH3A*, and *ABCB1*) were determined and participated in the construction of the prognostic signature. The Kaplan–Meier analysis of the 16 genes in the prognostic signature is shown in Supplementary Figure S6.

**TABLE 2:** Prognosis-related genes obtained from univariate Cox analysis.

Gene	HR	HR.95L	HR.95H	*p-v*alue
CCR7	0.757282	0.673764	0.851152	3.11E-06
ZAP70	0.720062	0.618221	0.83868	2.43E-05
FGD3	0.687978	0.574469	0.823914	4.80E-05
GZMM	0.756317	0.660316	0.866276	5.51E-05
CCL22	0.768989	0.676081	0.874663	6.38E-05
CD5	0.764502	0.669614	0.872837	7.14E-05
UBASH3A	0.682944	0.564518	0.826213	8.68E-05
NLRC3	0.660813	0.536951	0.813246	9.15E-05
CD6	0.75632	0.654665	0.87376	0.000149
FAM107A	0.699369	0.578115	0.846055	0.000233
TMC8	0.73798	0.627177	0.868358	0.000252
CD3E	0.803111	0.714091	0.903229	0.000254
PARP15	0.612672	0.468862	0.800591	0.000331
SPOCK2	0.821609	0.736946	0.915998	0.000398
MAP4K1	0.778105	0.677136	0.894128	0.000403
CD247	0.785414	0.686247	0.898911	0.000452
ABCB1	0.629333	0.485484	0.815804	0.00047
ITK	0.734542	0.61695	0.874548	0.000528
CD3D	0.836872	0.755412	0.927116	0.000654
IL2RG	0.817949	0.727	0.920275	0.000833
NAPSB	0.814394	0.721144	0.919702	0.000936
FCRL3	0.66462	0.521579	0.846888	0.000953
KLRB1	0.793575	0.691751	0.910386	0.000967
GRAP2	0.687556	0.550208	0.85919	0.000985
CD79A	0.882779	0.819572	0.95086	0.001004
PPP1R16B	0.784454	0.678319	0.907197	0.001064
MS4A1	0.799308	0.698678	0.914432	0.001103
CD19	0.809674	0.712222	0.92046	0.001252
BATF	0.79878	0.696769	0.915725	0.001269
CTSW	0.822953	0.730979	0.926499	0.001271
CCR4	0.777243	0.666568	0.906294	0.001303
TRAF3IP3	0.715535	0.583436	0.877544	0.001307
LGALS2	0.803134	0.70264	0.918001	0.001307
BLK	0.724381	0.592922	0.884988	0.001601
CLEC2D	0.730292	0.600642	0.887927	0.001622
CXCR3	0.825995	0.732821	0.931016	0.001745
LY9	0.674686	0.525527	0.866179	0.002022
CD27	0.851487	0.768029	0.944014	0.002253
SLAMF1	0.755151	0.629558	0.905799	0.002478
CD3G	0.800172	0.69193	0.925347	0.002645
WDFY4	0.767144	0.645377	0.911887	0.002648
CTLA4	0.796108	0.685818	0.924134	0.002727
SIRPG	0.808793	0.703203	0.930237	0.002948
GPR18	0.704885	0.559049	0.888766	0.003106
TIGIT	0.795028	0.682813	0.925686	0.00313
XCR1	0.711351	0.565779	0.894379	0.003551
RSF13B	0.703175	0.55466	0.891456	0.003624
FLT3LG	0.695071	0.543888	0.888277	0.003653
GIMAP7	0.825198	0.7249	0.939374	0.003662
ITGB7	0.784689	0.664842	0.92614	0.004139
P2RY10	0.802774	0.688598	0.935881	0.005007
SLAMF6	0.834623	0.734173	0.948818	0.005728
LYZ	0.901775	0.837561	0.970912	0.006085
IKZF3	0.836453	0.735118	0.951757	0.00672
CD28	0.770323	0.637652	0.930597	0.006814
IRF4	0.826852	0.720229	0.949259	0.00695
PDCD1	0.824498	0.716668	0.948551	0.006964
P2RY8	0.819641	0.706603	0.950762	0.008618
GIMAP1	0.774174	0.638544	0.938614	0.009198

**FIGURE 6:**
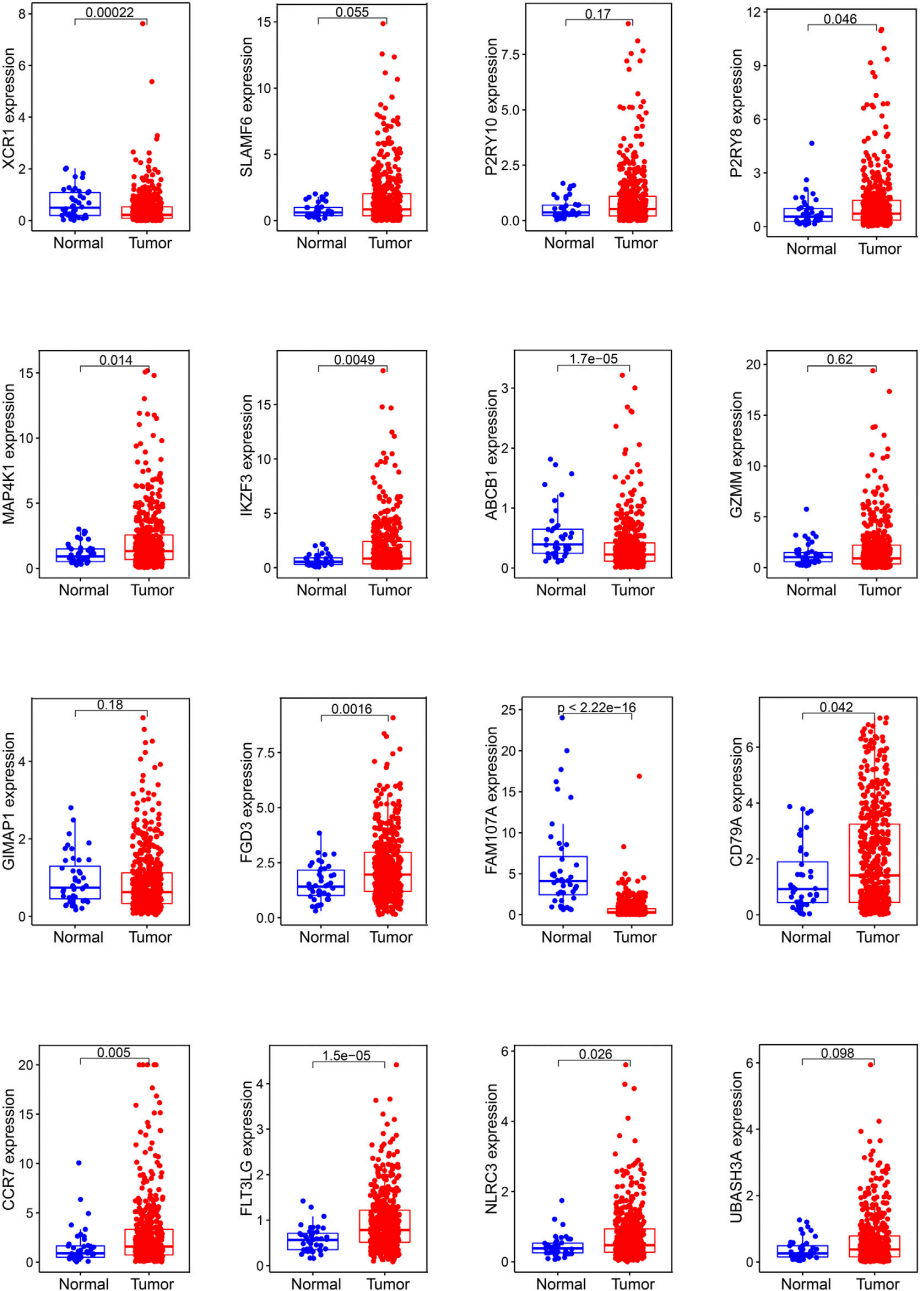
Expression of the 16 genes in the prognostic signature in normal and HNSCC samples.

**FIGURE 7:**
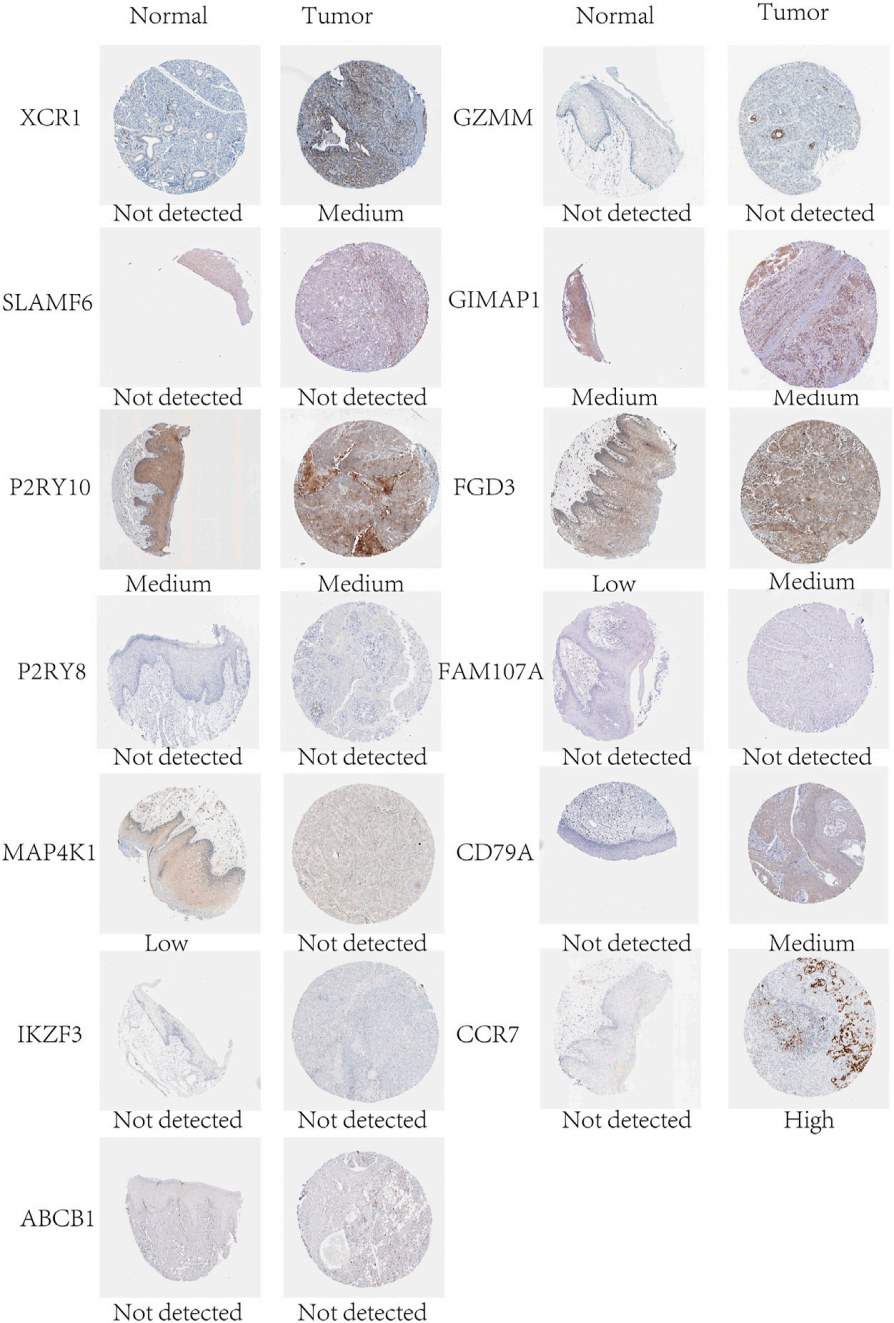
Protein expression levels of the genes of the prognostic signature in normal and HNSCC tissues in the HPA database.

In mRNA expression levels, *P2RY8*, *MAP4K1*, *IKZF3*, *FGD3*, *CD79A*, *CCR7*, *FLT3LG*, and *NLRC3* were highly expressed in HNSCC tissues. On the other hand, *XCR1*, *ABCB1*, and *FAM107A* were highly expressed in normal tissues (Figure 6). We further checked the protein levels of these prognostic genes between the HNSCC and normal tissues with the HPA database. Notably, we observed that the protein levels of *XCR1*, *FGD3*, and *CD79A* were moderately expressed in HNSCC tissues. The *CCR7* protein level was highly expressed in HNSCC tissues, and the protein levels of *P2RY10* and *GIMAP1* were both moderately expressed in HNSCC and normal tissues (Figure 7).

### Prognostic Signature and Immunity

Since the prognostic signature was developed based on TICI, we subsequently explored the potential relationship between the prognostic signature and immunity. Through correlation analysis between the prognostic genes in the gene signature and immune score, we observed that the 16 genes were all positively associated with the immune score (Supplementary Figure S7). The correlation analysis of the immune-infiltrating cells and risk score indicated that the infiltration of activated memory CD4^+^T cells, CD8^+^T cells, and follicular helper T cells and B cells was negatively related with the risk score, while the infiltration of M0 and M2 macrophages was positively related with the risk score (Supplementary Figure S8). Indeed, activated memory CD4^+^T cells, CD8^+^T cells, and follicular helper T cells and B cells had higher infiltration in the low-risk group, and M0 and M2 macrophages had higher infiltration in the high-risk group (Supplementary Figure S9A). Comparing the immune-related scores between the low- and high-risk groups, we found that the immune score and estimate score were higher in the low-risk group (Supplementary Figures S9B–D). The TIMER database was used to explore the relationship between the 16 prognostic genes and immune-infiltrating cells. As shown in Supplementary Figure S10, all the 16 genes (*P2RY8*, *FLT3LG*, *GIMAP1*, *CD79A*, *SLAMF6*, *FGD3*, *IKZF3*, *FAM107A*, *MAP4K1*, *GZMM*, *CCR7*, *P2RY10*, *XCR1*, *NLRC3*, *UBASH3A*, and *ABCB1*) were significantly associated with B cells, CD4^+^T cells, CD8^+^T cells, dendritic cells, neutrophils, and macrophages.

**FIGURE 8:**
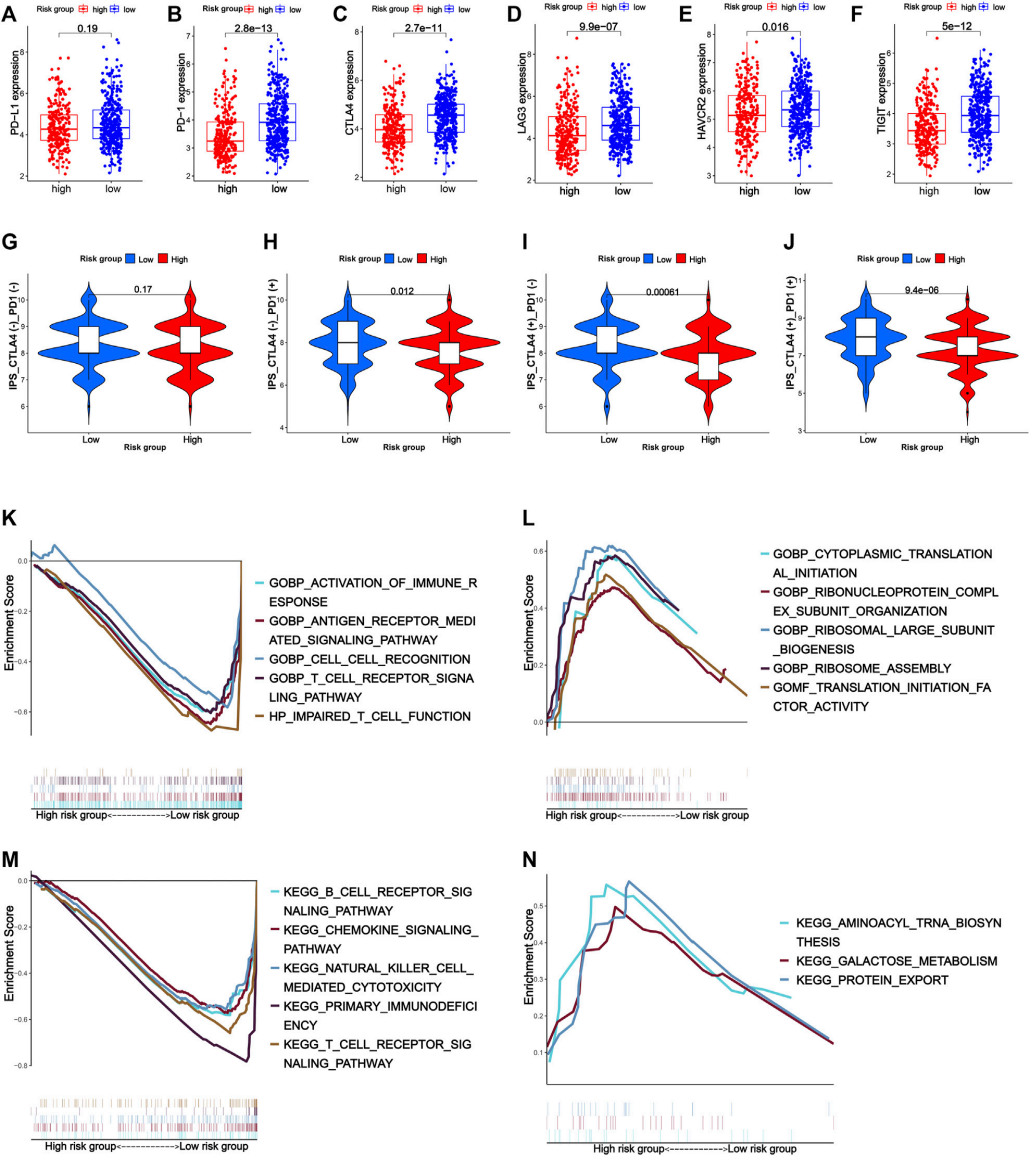
Expression of immune inhibitor receptors such as PD-L1 **(A)**, PD-1 **(B)**, CTLA-4 **(C)**, LAG3 **(D)**, HAVCR2 **(E)**, and TIGIT **(F)** between the high- and low-risk groups. **(G–J)** Response to immune checkpoint inhibitors of the high- and low-risk groups. **(K–N)** Gene set enrichment analysis. **(K)** Top five GO terms in the low-risk group. **(L)** Top five GO terms in the high-risk group. **(M)** Top five KEGG pathways in the low-risk group. **(N)** Top three KEGG pathways in the high-risk group.

Notably, the prognostic signature could distinguish the expression of immune inhibitor receptors (PD-1, CTLA-4, LAG3, HAVCR2, and TIGIT) and HLA genes well. Compared with the high-risk group, the low-risk group had high expression of PD-1, CTLA-4, LAG3, TIGIT, HAVCR2, and HLA genes (Supplementary Figure S9E). Unfortunately, there was no statistical difference in PD-L1 gene expression between the high- and low-risk groups (Figures 8A–F).

Considering the patients in the low-risk group had higher tumor immune cell infiltration and a higher expression of immune-related biomarkers, they were supposed to have better response to immunotherapy. As expected, the low-risk group had a higher IPS (immunophenoscore) of CTLA4 and PD-1, which reflected the percentages of the expression of certain immune genes on tumor-associated immune cells such as lymphocytes and macrophages and were biomarkers for good response to immune checkpoint inhibitor treatment, suggesting that the low-risk group might respond better to anti-PD-1 therapy, anti-CTLA-4 therapy, and anti-PD-1 combined with anti-CTLA-4 therapy (Figures 8G–J).

### Gene Set Enrichment Analysis

GSEA was used to explore the function and pathway enrichment of the high- and low-risk groups. In general, the low-risk group was associated with more immune-related GO terms and KEGG pathways compared with the high-risk group. GO analysis showed that the low-risk group was mainly related with activation of immune response, antigen receptor-mediated signaling pathway, cell–cell recognition, T-cell receptor signaling pathway, and impaired T-cell function. KEGG analysis indicated that the low-risk group was active in immune-associated pathways such as B-cell receptor signaling pathway, chemokine signaling pathway, natural killer cell-mediated cytotoxicity signaling pathway, primary immunodeficiency signaling pathway, and T-cell receptor signaling pathway, while there was little immune-related GO term and KEGG pathway enriched in the high-risk group (Figures 8K–N).

### Tumor Mutational Burden and Prognostic Signature

**FIGURE 9:**
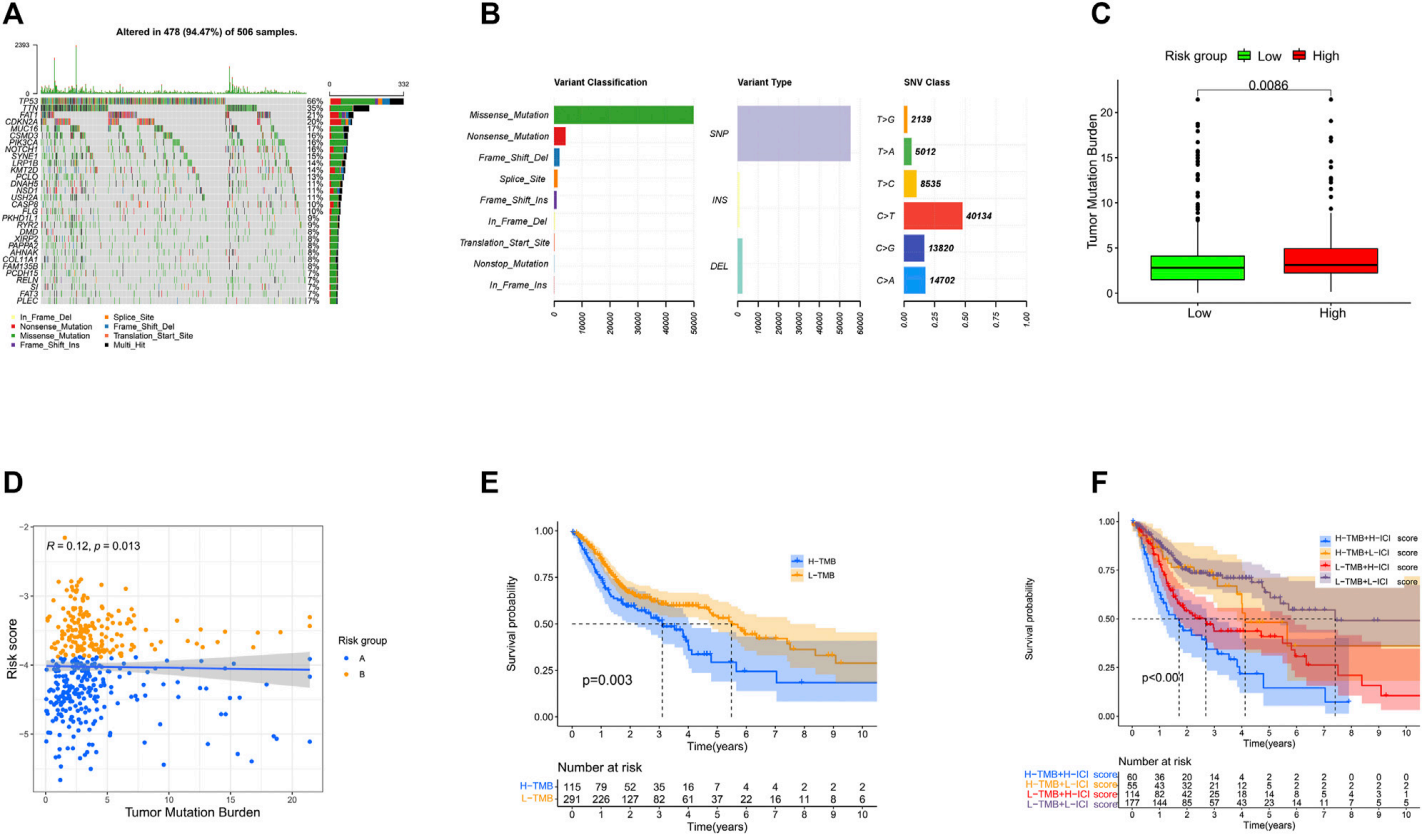
Landscape of the mutations in HNSCC. **(A)** Distribution of mutation types among the top 30 genes. **(B)** Variant classifications, variant types, and SNV classes. **(C)** Levels of tumor mutation burden in high- and low-risk groups. **(D)** Correlation between the risk score and tumor mutation burden. **(E)** Survival analysis of tumor burden mutation. **(F)** Survival analysis of tumor mutation burden combined with the risk score.

We downloaded the somatic mutation data of HNSCC patients from TCGA database and utilized the “maftools” package to visualize the mutation data. As illustrated in Figure 9, the top five mutated genes of HNSCC were TP53 (66%), TTN (35%), FAT1 (21%), CDKN2A (20%), and MUC16 (17%) (Figure 9A). Missense mutation, single-nucleotide polymorphism (SNP), and C > T were the most common mutation types in HNSCC (Figure 9B).

Then, we calculated the TMB (the total number of mutation events per million bases) of each HNSCC patient and explored the potential relationship of TMB and the prognostic signature. Interestingly, we found that the high-risk group had higher TMB, and there was a weak positive correlation between the TMB and risk score (Figures 9C,D). According to the optimal cutoff value (cutoff value being 4.2), the patients were classified into a high- (*n* = 115) or low-TMB (*n* = 291) group. The Kaplan–Meier analysis showed that the high-TMB group was associated with poorer prognosis (*p* = 0.003) (Figure 9E). Accordingly, the patients with a high TMB and high-risk score had the worst OS in our study (Figure 9F).

## Discussion

In the past few years, immune checkpoint inhibitors have reshaped the landscape of the treatment of HNSCC. The combined positive score (CPS) of PD-L1 was so far the most effective predictive biomarker of response to immunotherapy in HNSCC, while the other biomarkers including tumor mutation burden and microsatellite instability have not been well established. Extensive evidence indicates that tumor microenvironment including stromal cells, fibroblasts, extra-endothelial cells, innate immune cells (macrophages, neutrophils, dendritic cells, innate lymphocytes, bone marrow–derived suppressor cells, and NK cells), and adaptive immune cells (T cells and B cells) may be useful targets for immunotherapy strategies and closely related to the host’s antitumor ability (Ngiow et al., 2015; Srinivasan et al., 2018). Due to the importance and complexity of tumor immune cell infiltration, we aimed to explore the potential relationship between different immune cell infiltration patterns, HNSCC patients’ prognosis, and immune heterogeneity and to identify prognosis-related DEGs by comparing the expression profiles of different infiltration patterns. Finally, we developed a TICI-based 16-gene prognostic signature.

First, we preliminarily explored the relationship between tumor immune-infiltrating cells and prognosis. It is well known that NK cells and CD8^+^T cells are the most promising targets in immunity therapy. NK cells directly induce tumor cell death by releasing perforins and granzymes, and cytotoxic CD8^+^T cells induce tumor cell death with the engaging of the major histocompatibility complex (MHC) with or without the help of Th cells (Cheng et al., 2013; Henning et al., 2018). Other important cells such as macrophages accumulate significantly in tumor microenvironment. M1 macrophages secrete pro-inflammatory cytokines and have antitumor properties, while M2 macrophages produce anti-inflammatory cytokines and exert pro-tumor properties (Xiao et al., 2020). In our study, immune-infiltrating T cells and B cells were associated with superior prognosis, while M0 macrophages, neutrophils, and mast cells were related with poor prognosis. Using consensus clustering, we obtained two immune cell infiltration patterns. The cluster with high infiltrating cells such as T cells, B cells, NK cells, and M1 macrophages was named the high-TICI group. On the other hand, the cluster with high infiltration of immune resting cells or inflammatory cells such as resting memory CD4^+^T, resting NK cells, M0 macrophages, neutrophils, and mast cells was named the low-TICI group. The heterogeneity of tumor immune cell infiltration was also a vital factor in determining the patients’ prognosis. Zeng et al. (2019) found that high tumor-infiltrating CD4^+^T cells, CD8^+^T cells, NK cells, and M1 macrophages were associated with favorable prognosis in gastric cancer. Just as the previous study, the high-TICI group with high infiltration of T cells, B cells, NK cells, and M1 macrophages was related to improved OS, and the low-TICI group with low infiltration of these cells was related to poor OS.

Except for difference in immune cell infiltration, the high-TICI and low-TICI groups had significant difference in immune score, estimate score, HLA gene, and T-cell activation-related receptor (PD-L1, PD-1, CD28, CD80, CD86) expression. In detail, the high-TICI group had higher immune score, estimate score, and higher expression of HLA genes and T-cell activation-related genes. Extensive studies showed that the expression of the immunosuppressive receptors negatively regulated T cells; however, contradictory to the aforementioned mechanism was that high immune cell infiltration, especially high T-cell infiltration, was accompanied by a high expression of immune inhibitor receptors. One possible explanation could be that the increased immune infiltrating cells triggered antitumor immunity and escaped the antitumor immunity; the tumors upregulated the expression of these immunosuppressive genes such as PD-L1 expression (Klümper et al., 2020; Liu et al., 2020). In turn, the immune-infiltrating cells were downregulated by immune inhibitor receptors (Juneja et al., 2017). Under these circumstances, treating with immune checkpoint inhibitors would bring the patients’ best response to immunotherapy. Thus, immune inhibitor genes and tumor immune cell infiltration used together to evaluate the response to immunotherapy could be more effective.

Using weighted gene co-expression network analysis, we acquired 408 TICI-related co-expressed genes. Different patterns of immune infiltration generated different gene expression characteristics. In our study, mRNAs related to the functions of T-cell activation, leukocyte proliferation, lymphocyte differentiation and proliferation, and MHC class II receptor activity were highly expressed in the high-TICI group, whereas they were low expressed in the low-TICI group. Similarly, mRNAs correlated with the signaling pathways of hematopoietic cell lineage, intestinal immune network for IgA production, cell adhesion molecules, and Th1, Th2, and Th17 cell differentiation were highly expressed in the high-TICI group and low expressed in the low-TICI group.

With multivariate Cox analysis, we developed a 16-gene prognostic signature. Almost all the 16 prognostic genes (*P2RY8*, *P2RY10*, *FLT3LG*, *GIMAP1*, *CD79A*, *SLAMF6*, *FGD3*, *IKZF3*, *FAM107A*, *MAP4K1*, *GZMM*, *CCR7*, *XCR1*, *NLRC3*, *UBASH3A*, and *ABCB1*) were immune-related. *GZMM* (granzyme) was an exogenous serine protease highly expressed in NK cells and cytotoxic lymphocytes. *GZMM* could induce different cell deaths by activating apoptosis-related enzyme systems. Wang et al. (2012) showed that the human NK cell line KHYG-1 with high *GZMM* expression showed great ability to kill tumor cells. *CCR7* (C-C Motif Chemokine Receptor 7) was a protein-coding gene and its encoded protein was a member of the G protein–coupled receptor family. Itakura et al. (2013) proved that *CCR7* played a unique role in regulating T-cell activation and controlling the migration of memory T cells to inflamed tissues. *CCR7* expression was associated with better prognosis in lung cancers. *P2RY8* (P2Y Receptor Family Member 8) was a Gα13-coupled receptor, which regulated the migration inhibition and growth of B cells. *P2RY8* was frequently mutated in diffuse large B-cell lymphoma and Burkitt lymphoma (Lu et al., 2019; He et al., 2022). *P2RY10* (P2Y Receptor Family Member 10) was also a G-protein-coupled receptor. *P2RY10* could facilitate chemokine-induced CD4^+^T cell migration and was potentially involved in the immune response (Gurusamy et al., 2021). *FLT3LG* (Fms Related Receptor Tyrosine Kinase 3 Ligand) combined with *FLT3* on dendritic cells to stimulate their differentiation and proliferation. In addition, *FLT3LG* was a biomarker that reflected the clinical response of oxaliplatin (Pol et al., 2020). *GIMAP1* (GTPase, IMAP Family Member 1) was involved in the differentiation of Th cells and was essential for the development and survival of mature B and T lymphocytes (Saunders et al., 2010). *CD79A* was a B cell receptor; Tagliabue et al. (2020) found that high expression of *CD79A* was associated with significantly better prognosis of laryngeal cancer. *SLAMF6* was an immune inhibitor receptor, and its overexpression led to the exhaustion and decreased proliferation ability of CD8^+^T cells (Yigit et al., 2019; Hajaj et al., 2020). *FGD3* was a protein-coding gene, and Susini et al. (2021) found that high expression of FGD3 in breast cancer was associated with good disease-free survival and overall survival. Moreover, it was also a prognosis-related gene of head and neck squamous cell carcinoma, lung adenocarcinoma, cervical squamous cell carcinoma, bladder urothelial carcinoma, and sarcoma. *IKZF3* was a specific transcription factor involved in regulating lymphocyte proliferation and differentiation. Highly expressed *IKZF3* in T cells was related with improved OS in symptomatic stage III multiple myeloma patients treated with immunotherapy (Awwad et al., 2018). *FAM107A* (Family with sequence similarity 107, member A) was a candidate tumor suppressor gene. It was low expressed in laryngeal squamous cell carcinoma and participated in the occurrence of lung cancer (Zhou et al., 2020; Yan and Chen, 2021). *MAP4K1* (Mitogen-Activated Protein Kinase Kinase Kinase Kinase 1) was involved in promoting T-cell failure in multiple cancers, while it was associated with favorable prognosis of muscle-invasive bladder cancer (van der Heijden et al., 2016). *XCR1* was a chemokine receptor of the G protein-coupled receptor superfamily. A study showed that cross-presenting *XCR1* dendritic cells had a specialized ability to initiate effector CD8^+^T cells and mediate antitumor responses. Therefore, *XCR1* could be considered a target for cancer immunotherapy (Audsley et al., 2020). *NLRC3* (NLR Family CARD Domain Containing 3) was negatively regulating the innate immune response. In addition, *NLRC3* could attenuate PI3K-mTOR signaling pathways and inhibit colorectal cancer cell proliferation (Karki et al., 2016). *UBASH3A* (Ubiquitin-associated and SH3 containing A) was a negative regulator of T cells. Combined with CBL-B, *UBASH3A* could inhibit CD28-mediated signal transduction, thereby negatively regulating T-cell activation. *UBASH3A* also played an important role in autoimmunity (Ge et al., 2019). *ABCB1* (ATP Binding Cassette Subfamily B Member 1) was shown to involve in multiple chemotherapeutic drug resistance (Zhou et al., 2019; Belisario et al., 2020; Luo et al., 2020).

Importantly, the prognostic signature constructed by the aforementioned 16 genes could distinguish HNSCC patients with favorable and poor prognosis, and it could even distinguish the prognosis of HNSCC patients with different clinical characteristics well. ROC curves and OS nomogram further confirmed the sensitivity and accuracy of the prognostic signature. In addition, two subgroups (TCGA-HNSCC cohort and GSE65858 cohort) and an independent dataset (GSE41613 cohort) achieved a similar prognostic significance.

In addition, tumor immune cell infiltration levels and immune inhibitor receptor expression were also significantly different in high- and low-risk groups. Specifically, the low-risk group had higher infiltration of activated memory CD4^+^T cells, CD8^+^T cells, and follicular helper T cells and B cells, and had higher expression of PD-1, CTLA-4, LAG3, TIGIT, and HAVCR2. CTLA-4, LAG3, TIGIT, and HAVCR2 had a synergistic effect with PD-L1 in negatively regulating T cells, adaptive, or innate immunity. Clinical studies have shown that dual blockade of PD-1 and CTLA-4/LAG3/TIGIT/HAVCR2 enhanced the proliferation and function of CD8^+^T cells and tumor-infiltrating lymphocytes as compared with single blockade and could provide survival benefit as compared with PD-L1 blockade alone for patients with PD-L1-positive cancers (Chauvin et al., 2015; Andrews et al., 2017; Chauvin and Zarour, 2020; Rodriguez-Abreu et al., 2020; Trüb et al., 2020). Since the prognostic signature distinguished the expression of these promising immunotherapeutic targets well, it might be able to reflect the response to immune checkpoint inhibitor treatment. Consistent with our guess, the low-risk group had a higher IPS of PD-L1, CTLA-4, and PD-L1-CTLA-4, which indicated that the low-risk group might respond better in anti-PD-L1 therapy, anti-CTLA-4 therapy, and anti-PD-L1 combined with anti-CTLA-4 therapy.

Accumulated studies have shown that high tumor mutational burden (TMB) is associated with better response to immune checkpoint inhibitor treatment. Subsequently, we further analyzed the mutational landscape of HNSCC. TP53 mutation is the most common, accounting for 66% of all mutation types in HNSCC and up to 75% in non-HPV-related HNSCC (Zhou et al., 2016; Klinakis and Rampias, 2020). TP53 is a tumor suppressor gene, which is mainly involved in mediating the cellular stress response after DNA damage and maintaining the stability of genetic material. Mutated TP53 is a proto-oncogene and could promote cell malignant transformation. Interestingly, the correlation between TP53 mutation, immunotherapy response, and patients’ prognosis was cancer-type dependent. Chen et al. (2019) found that non–small cell lung cancer (NSCLC) patients with high TMB levels, mainly TP53 mutation, benefited significantly from immune checkpoint inhibitor (Ozaki et al., 2020), while the immunotherapy response of patients with TP53 wild-type is better than that of TP53 mutation. Patients with TP53 mutation had poor OS with immune checkpoint inhibitor therapy in colon adenocarcinoma (COAD) and gastrointestinal tumors (GI) (Li et al., 2020). In HNSCC, TP53 mutations have been proven to be associated with decreased immune cell infiltration, low PD-L1 expression, and poor prognosis (Klinakis and Rampias, 2020; Li et al., 2020). Surprisingly, and perhaps reasonably, we found that the high-risk group had higher TMB, and there was a weak positive correlation between the TMB and risk score. In addition, high TMB was associated with poorer prognosis of HNSCC patients. Nevertheless, the exact mechanism of the TMB and HNSCC patients’ prognosis as well as the mechanism of TMB and immunotherapy response still needs to be further explored.

There are some advantages to our study. First of all, to our knowledge, this is the first study to develop a TICI-related prognostic signature composed of 16 genes for HNSCC. Second, our prognostic model is closely relevant to immune-related scores (IPS, immune score, and estimate score), the expression of immune inhibitor receptors (PD-1, CTLA-4, LAG3, TIGIT, HAVCR2), and immune-related pathways. These results, together with tumor mutation burden, might provide some helpful information for the development of new immunotherapeutic and prognostic biomarkers. Moreover, by comparing the 16 mRNAs and their protein expression levels of normal and tumor tissues from the public database, it might directly or indirectly suggest the role of these genes and proteins in the development of head and neck tumors. Finally, the 16-gene prognostic signature was validated in TCGA-HNSCC, GSE65858, and GSE41613 cohorts. However, *in vivo* and *in vitro* experiments are needed to further explore the mechanisms on how the 16 genes and prognostic signature impact immunity and prognosis of HNSCC.

Immunotherapy is the new pillar of antitumor treatment of HNSCC. It produces memory CD8^+^T cells, which have long-lasting protection and effectively prevent metastasis and recurrence. However, the biomarkers of immunotherapy are still being explored. It is believed that in the near future there will be a more complete immunotherapy system to better guide the immunotherapy and improve the prognosis of cancer patients.

## Conclusion

In conclusion, this study developed a TICI-based 16-gene prognostic signature for HNSCC, and the 16 genes might be potential immunotherapeutic and prognostic biomarkers.

## Data Availability

The datasets presented in this study can be found in online repositories. The names of the repository/repositories and accession number(s) can be found in the article/Supplementary Material.
