# Supplementary figures and images for: Immune Infiltration Characteristics and a Gene Prognostic Signature Associated With the Immune Infiltration in Head and Neck Squamous Cell Carcinoma
Source: Front Genet. 2022 May 2;13:848841. doi: 10.3389/fgene.2022.848841 (PMC9108548; doi:10.3389/fgene.2022.848841)

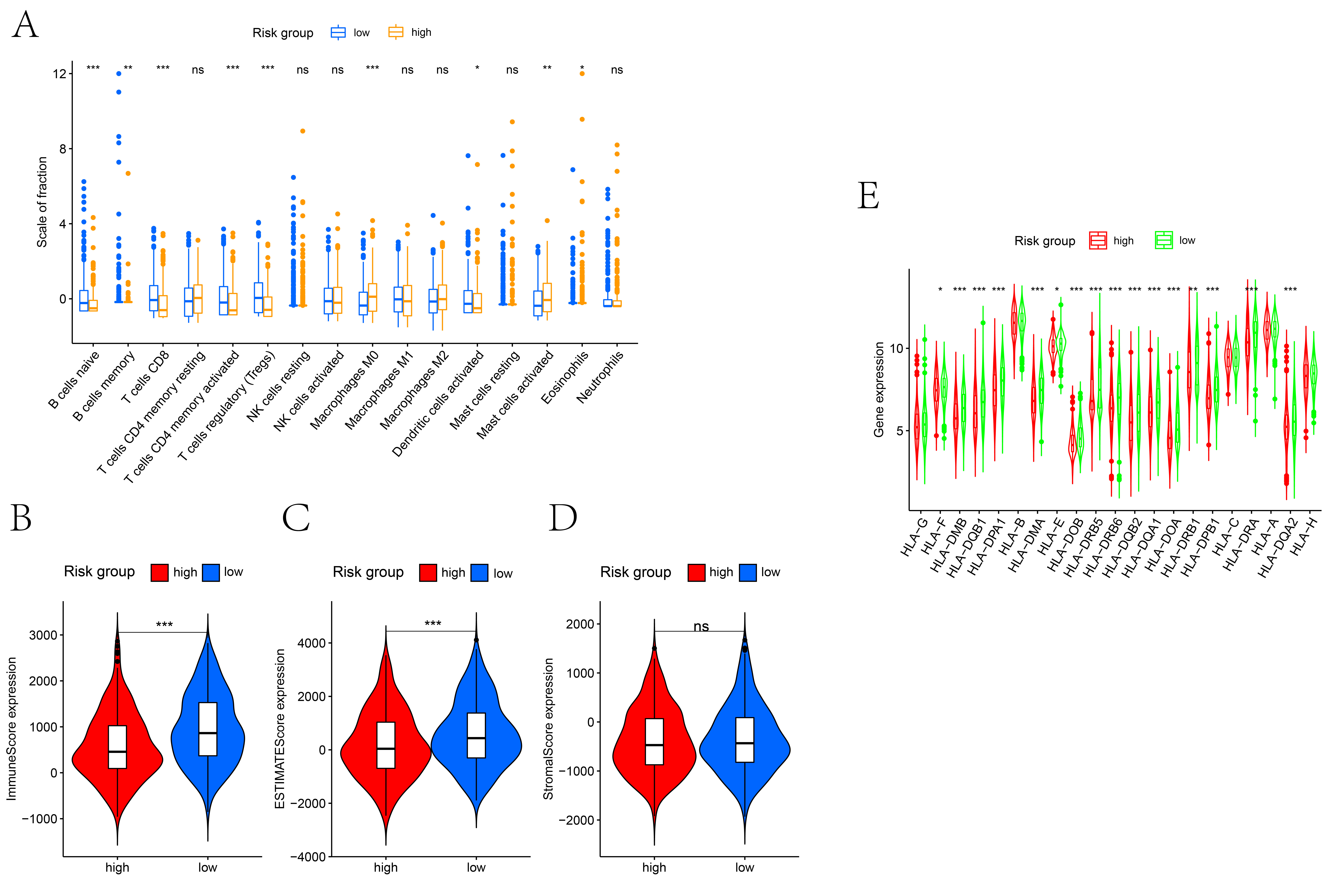

Supplement: Supplementary file 1 [file DataSheet3.ZIP › revisedSupplementaryMaterials3/revisedFigure S9.jpg]

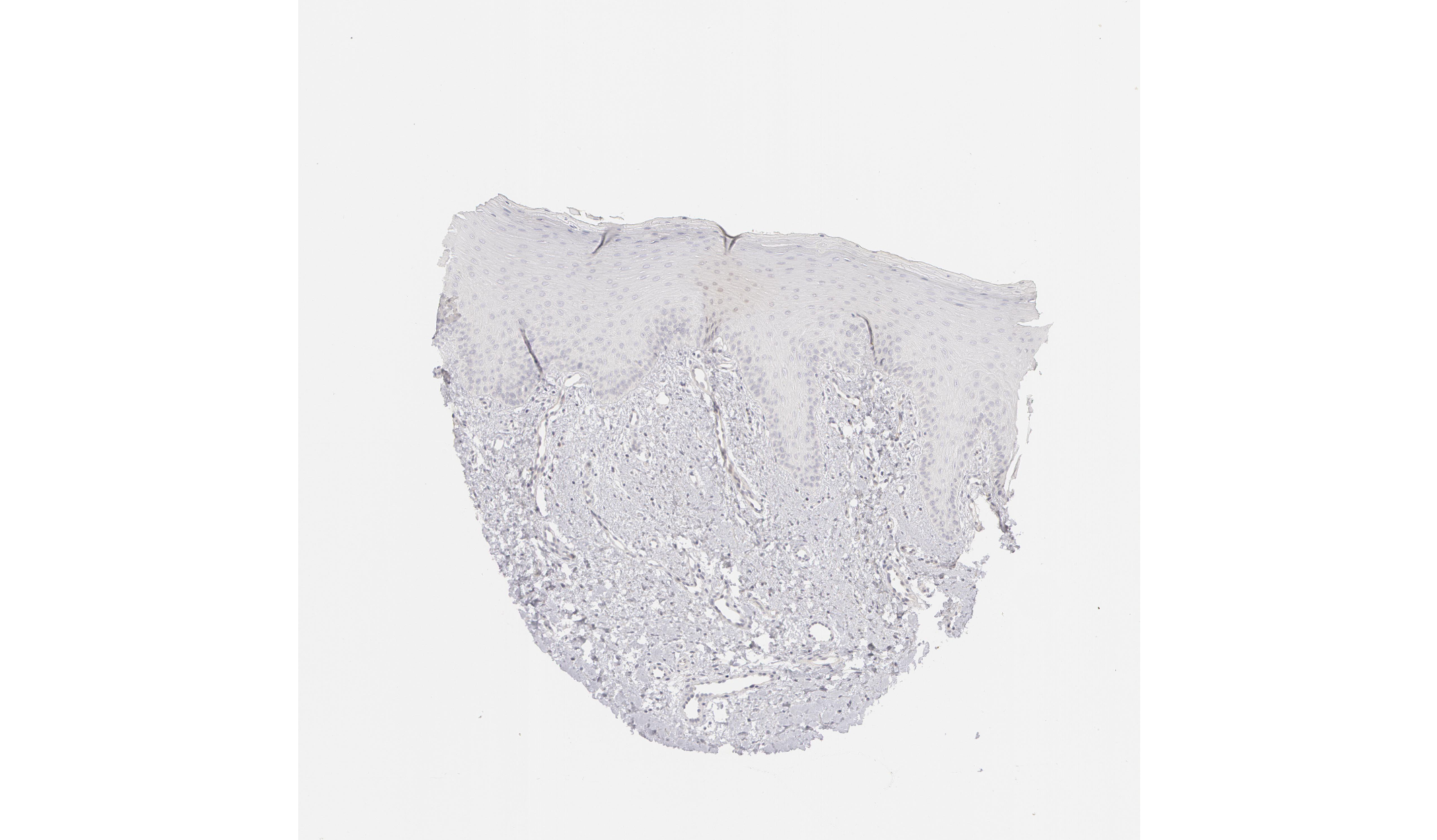

Supplement: Supplementary file 2 [file DataSheet4.ZIP › Immunohistochemistry(1)/ABCB1Normal.png]

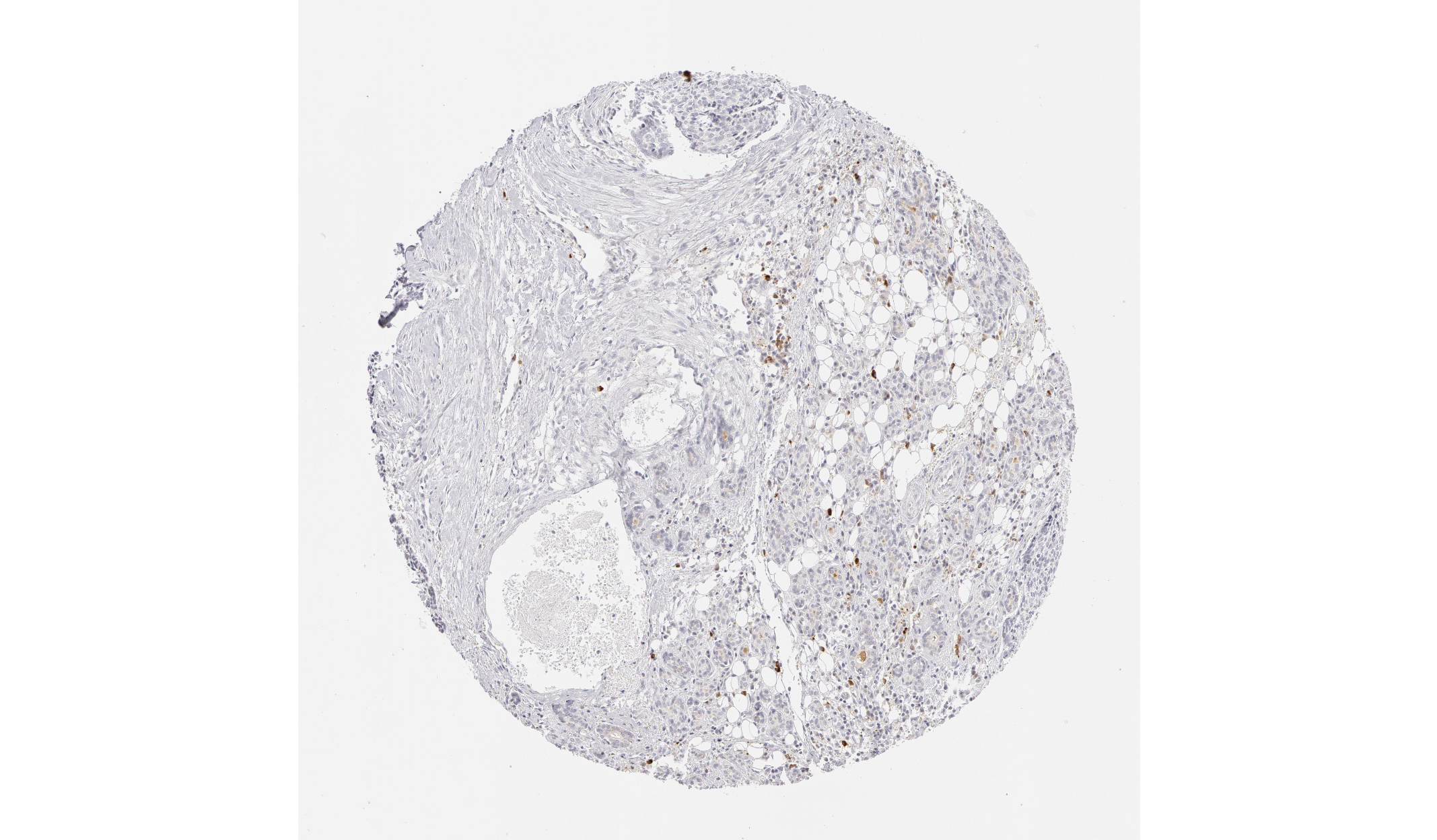

Supplement: Supplementary file 2 [file DataSheet4.ZIP › Immunohistochemistry(1)/ABCB1Tumor.png]

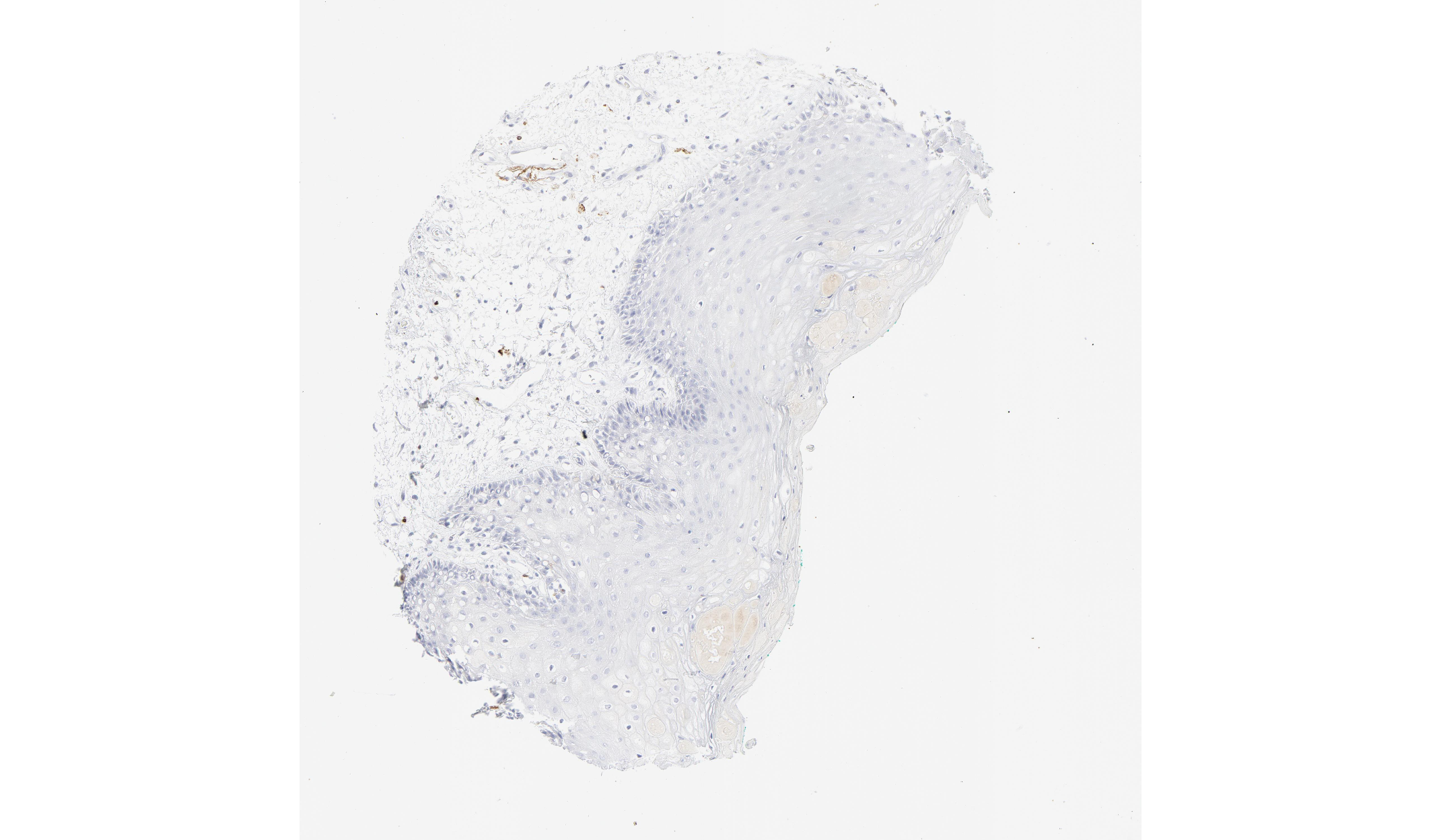

Supplement: Supplementary file 2 [file DataSheet4.ZIP › Immunohistochemistry(1)/CCR7Normal.png]

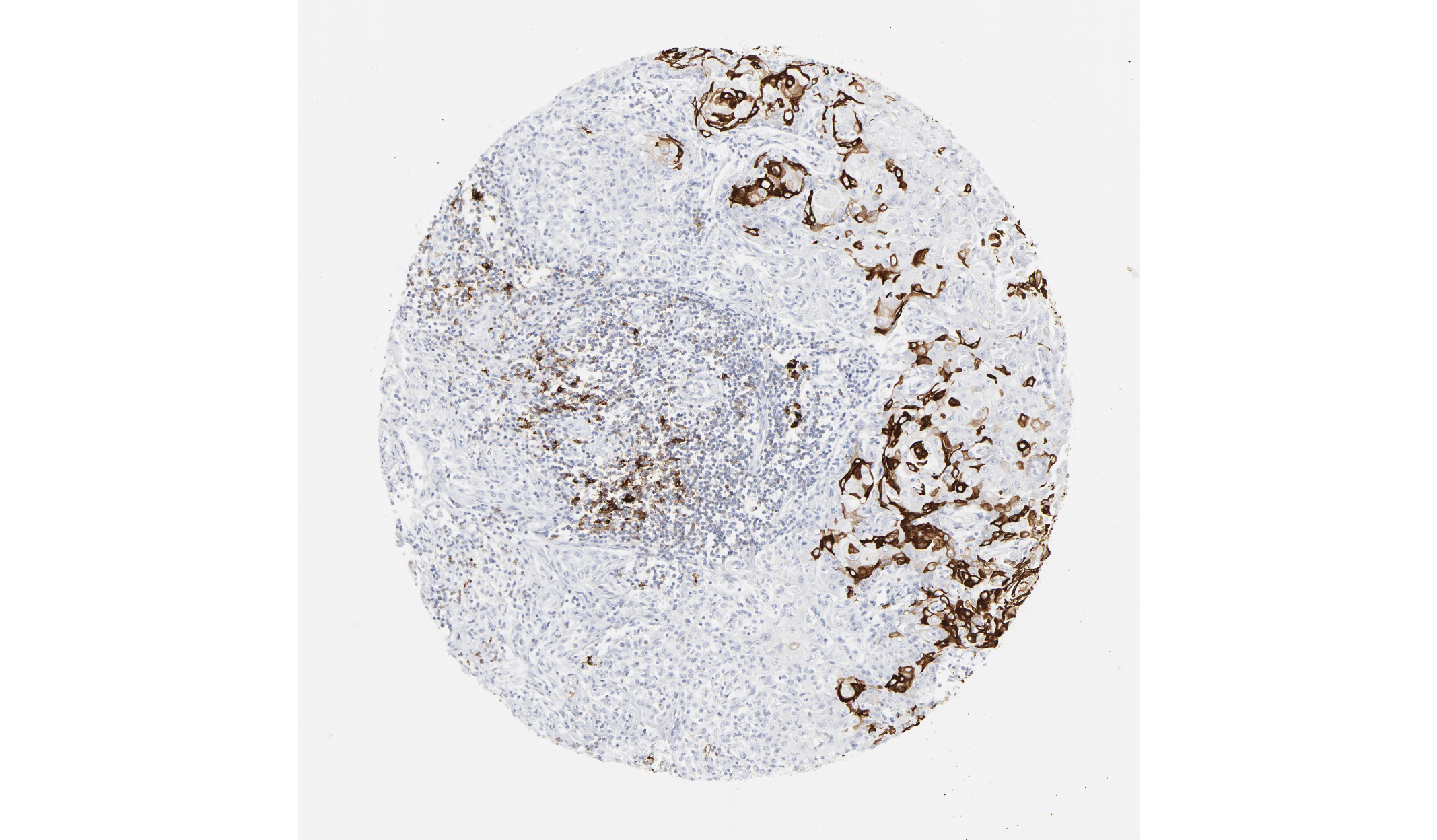

Supplement: Supplementary file 2 [file DataSheet4.ZIP › Immunohistochemistry(1)/CCR7Tumor.png]

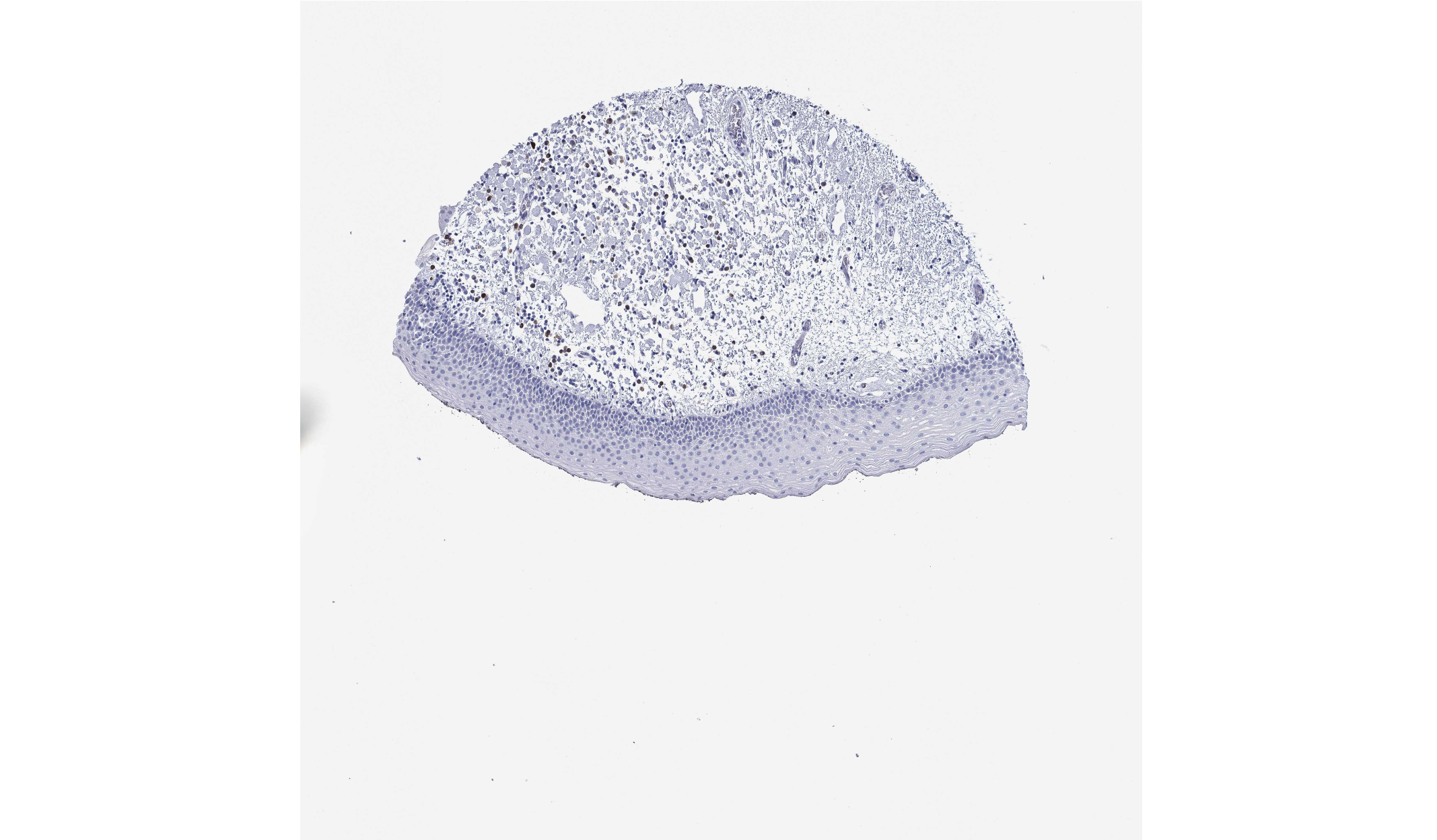

Supplement: Supplementary file 2 [file DataSheet4.ZIP › Immunohistochemistry(1)/CD79ANormal.png]

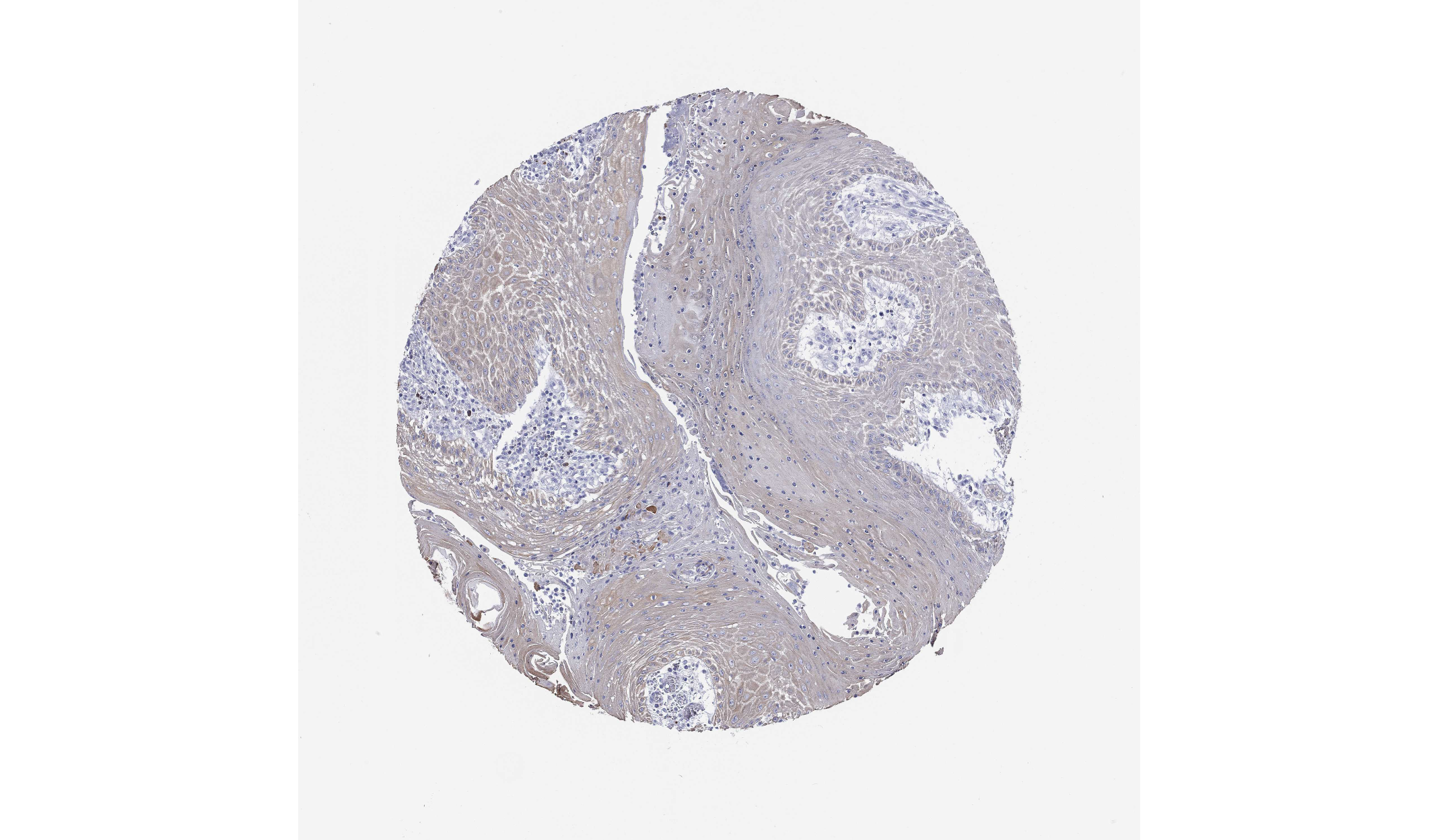

Supplement: Supplementary file 2 [file DataSheet4.ZIP › Immunohistochemistry(1)/CD79ATumor.png]

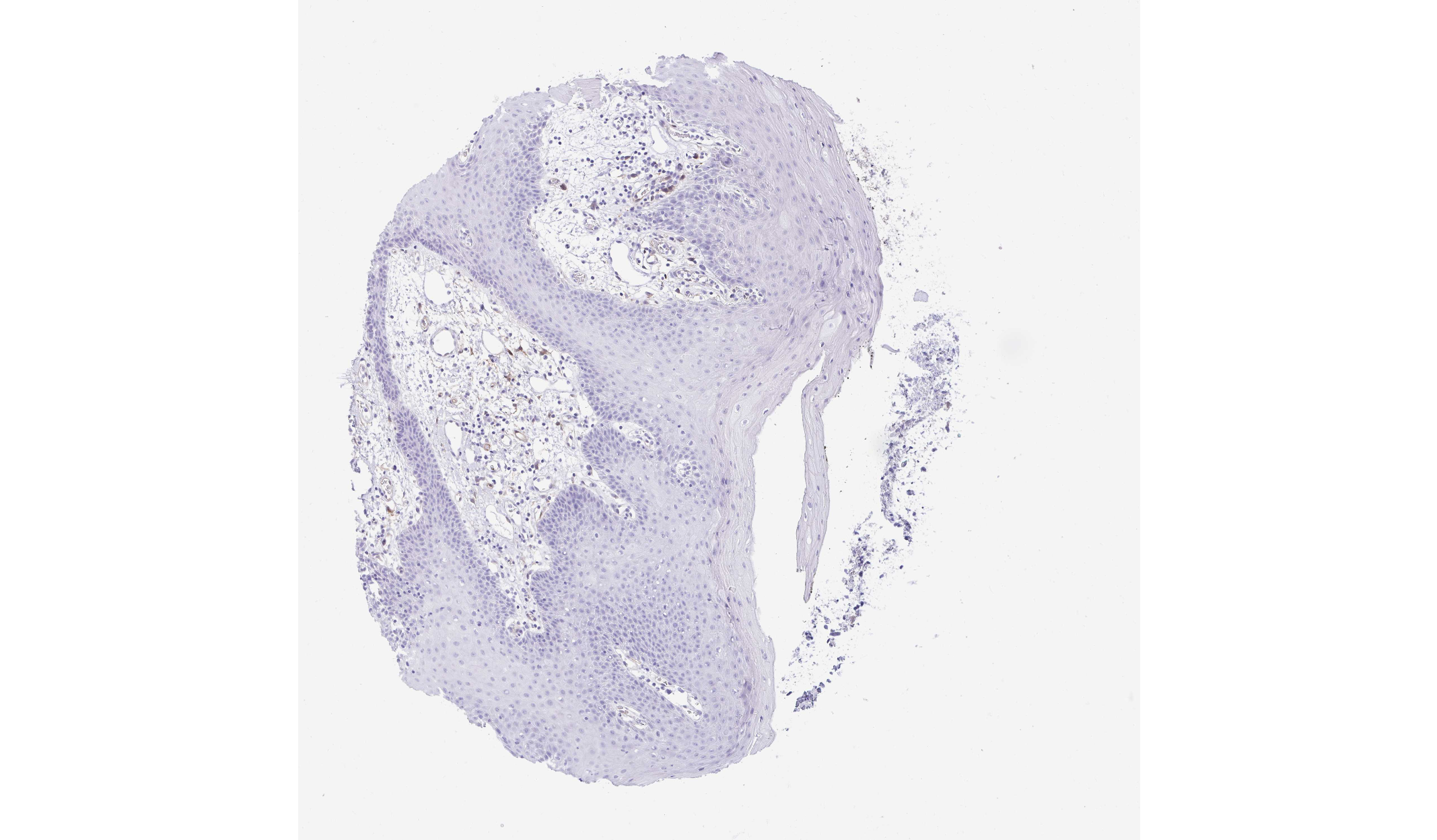

Supplement: Supplementary file 2 [file DataSheet4.ZIP › Immunohistochemistry(1)/FAM107ANormal.png]

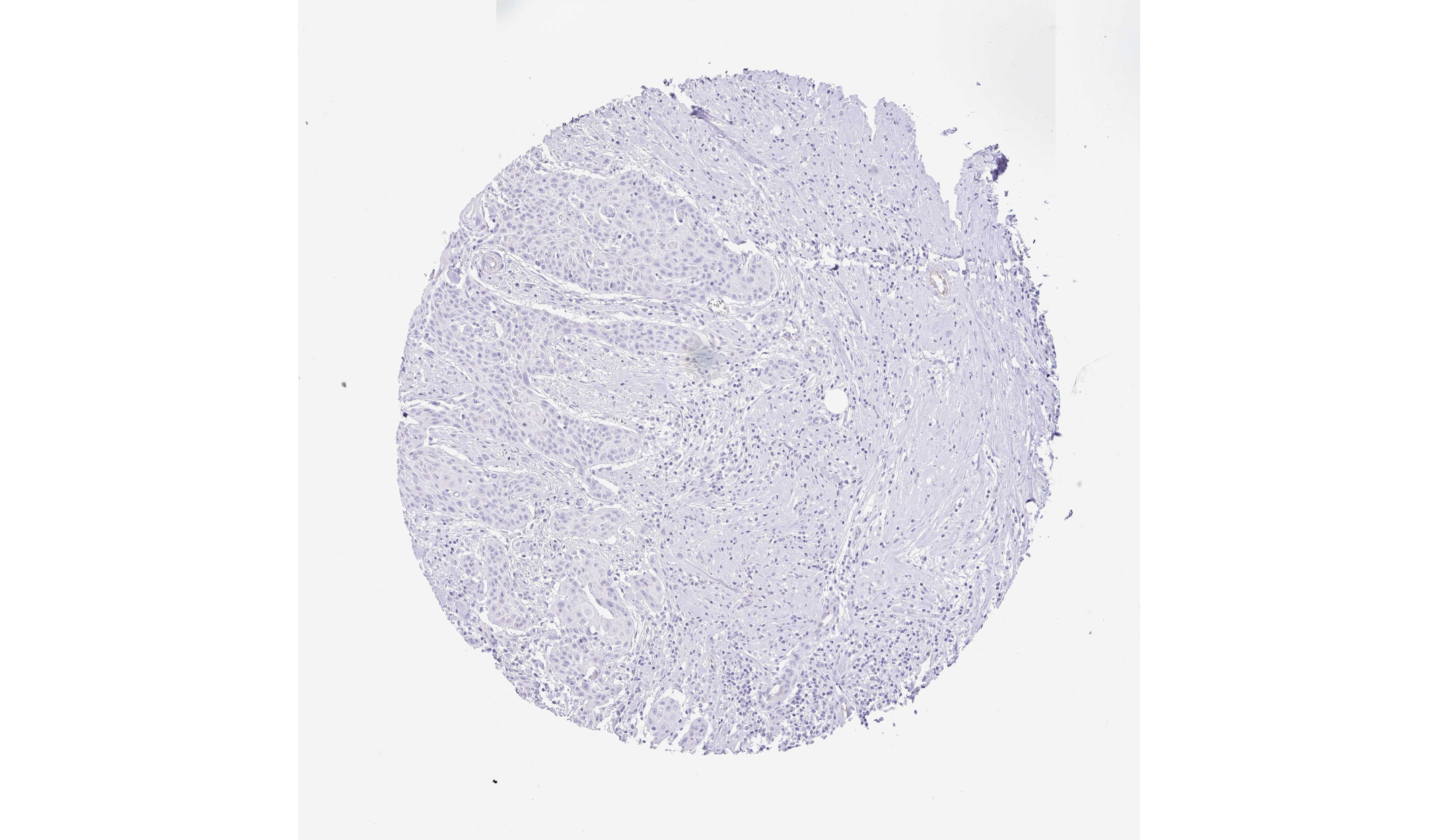

Supplement: Supplementary file 2 [file DataSheet4.ZIP › Immunohistochemistry(1)/FAM107ATumor.png]

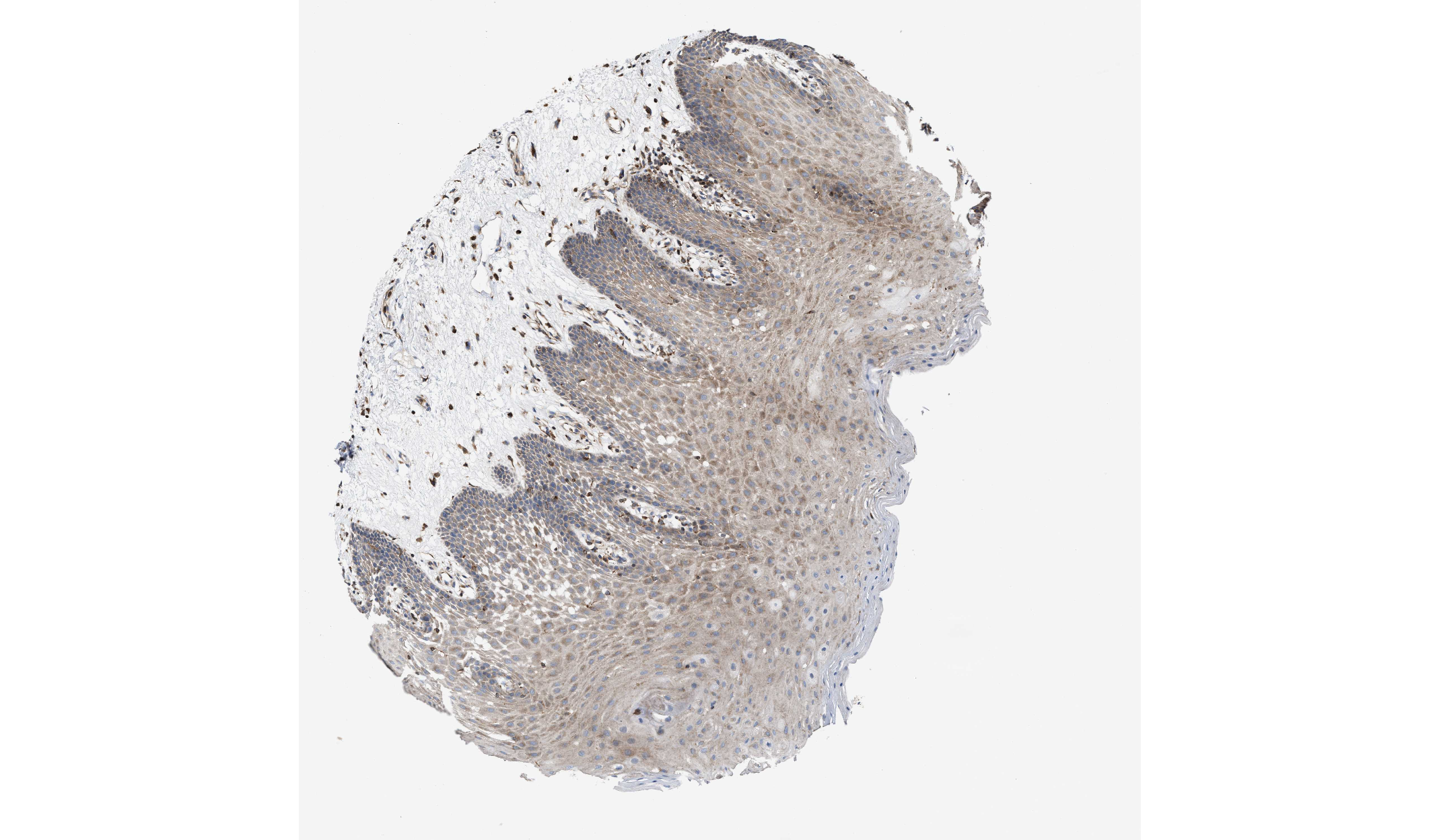

Supplement: Supplementary file 2 [file DataSheet4.ZIP › Immunohistochemistry(1)/FGD3Normal.png]

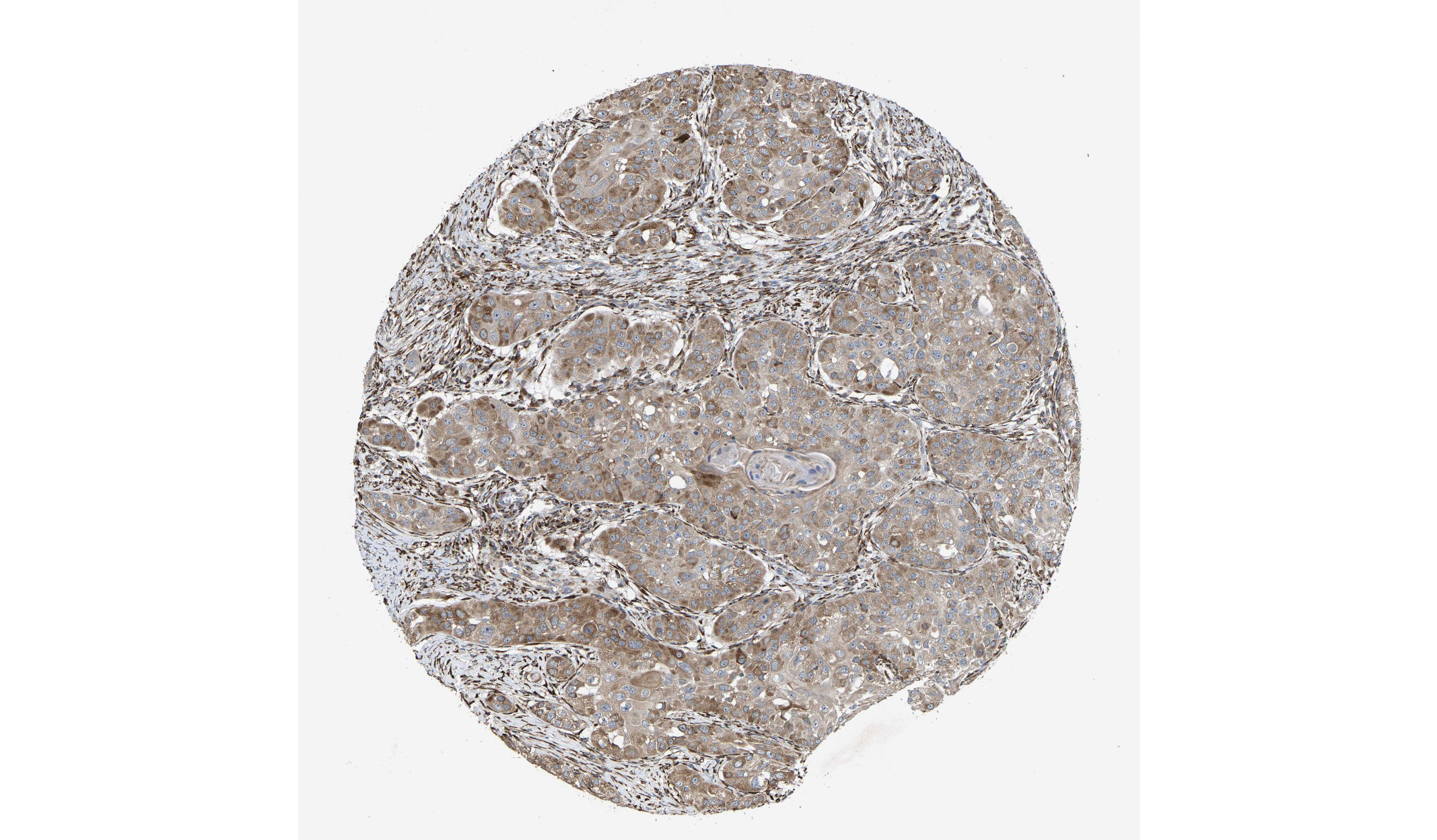

Supplement: Supplementary file 2 [file DataSheet4.ZIP › Immunohistochemistry(1)/FGD3Tumor.png]

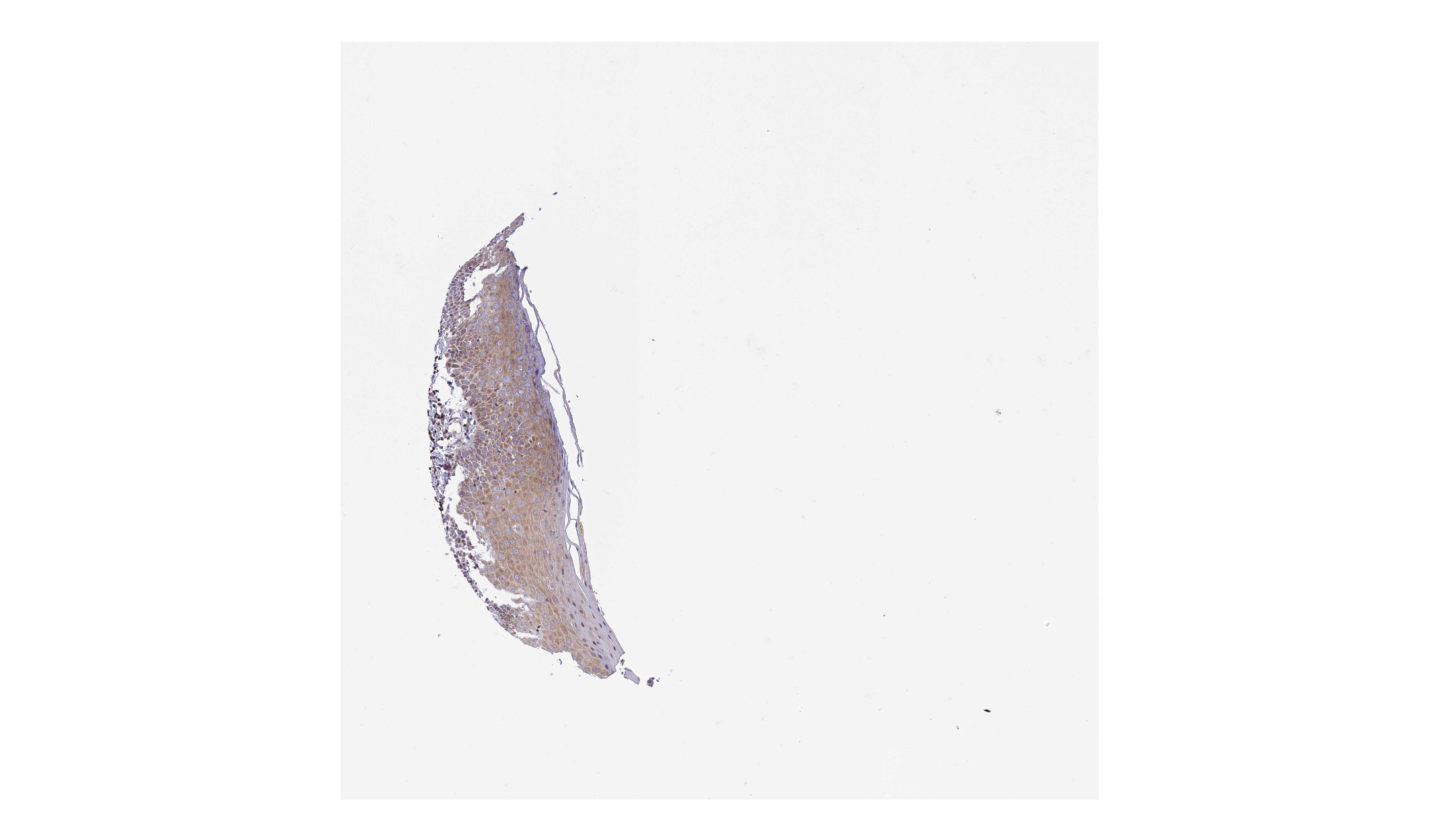

Supplement: Supplementary file 2 [file DataSheet4.ZIP › Immunohistochemistry(1)/GIMAP1Normal.png]

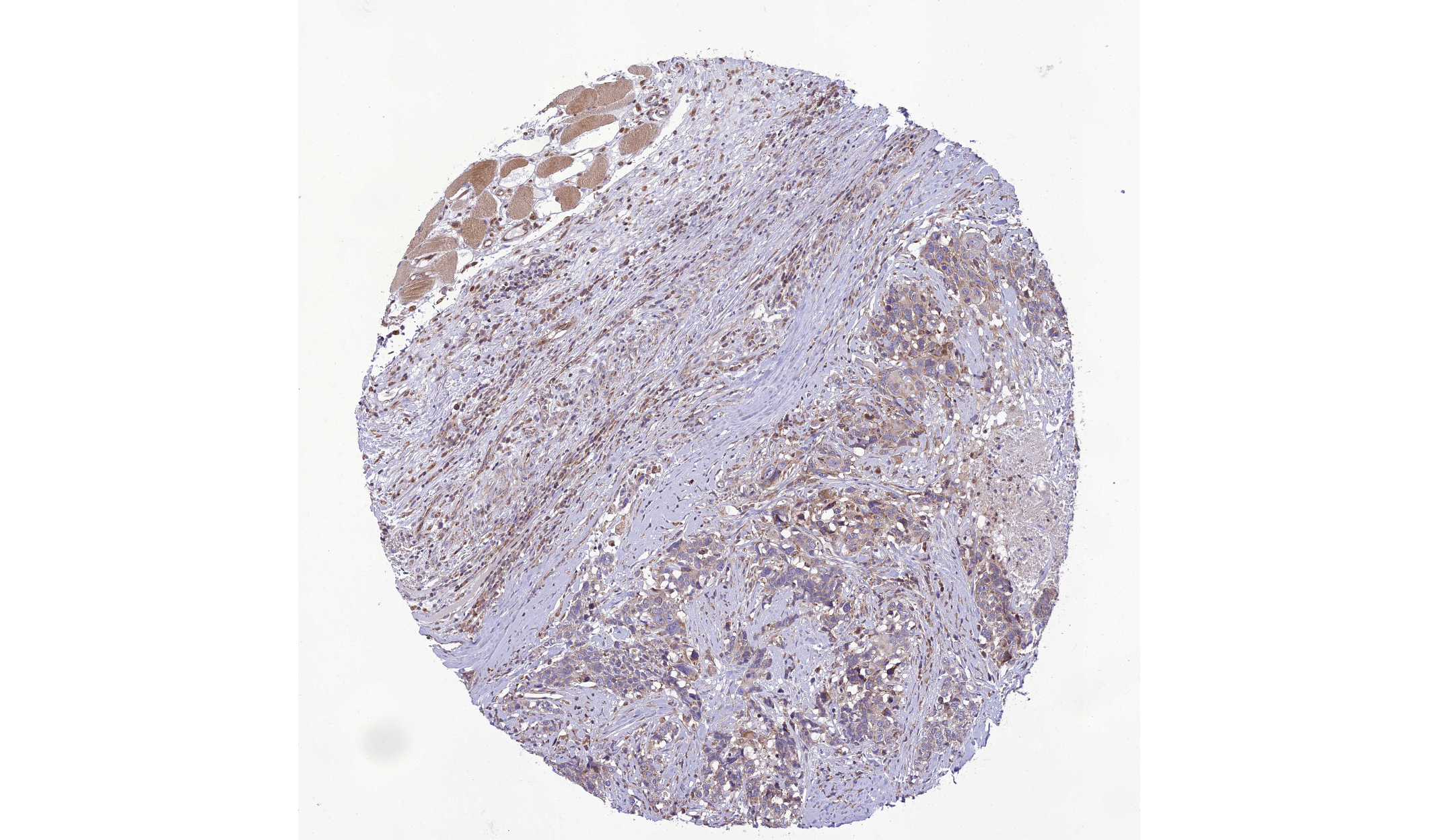

Supplement: Supplementary file 2 [file DataSheet4.ZIP › Immunohistochemistry(1)/GIMAP1Tumor.png]

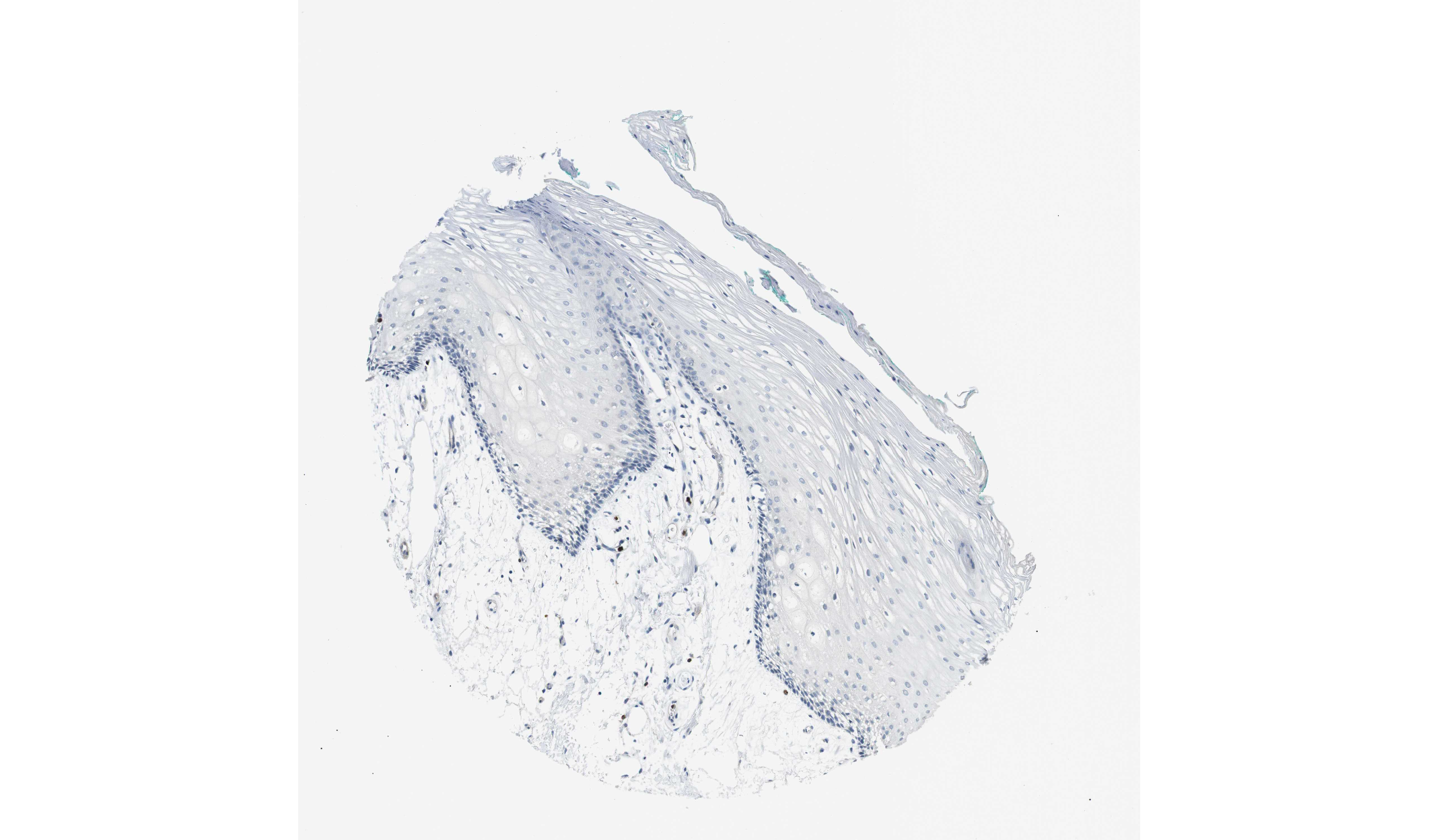

Supplement: Supplementary file 2 [file DataSheet4.ZIP › Immunohistochemistry(1)/GZMMNormal.png]

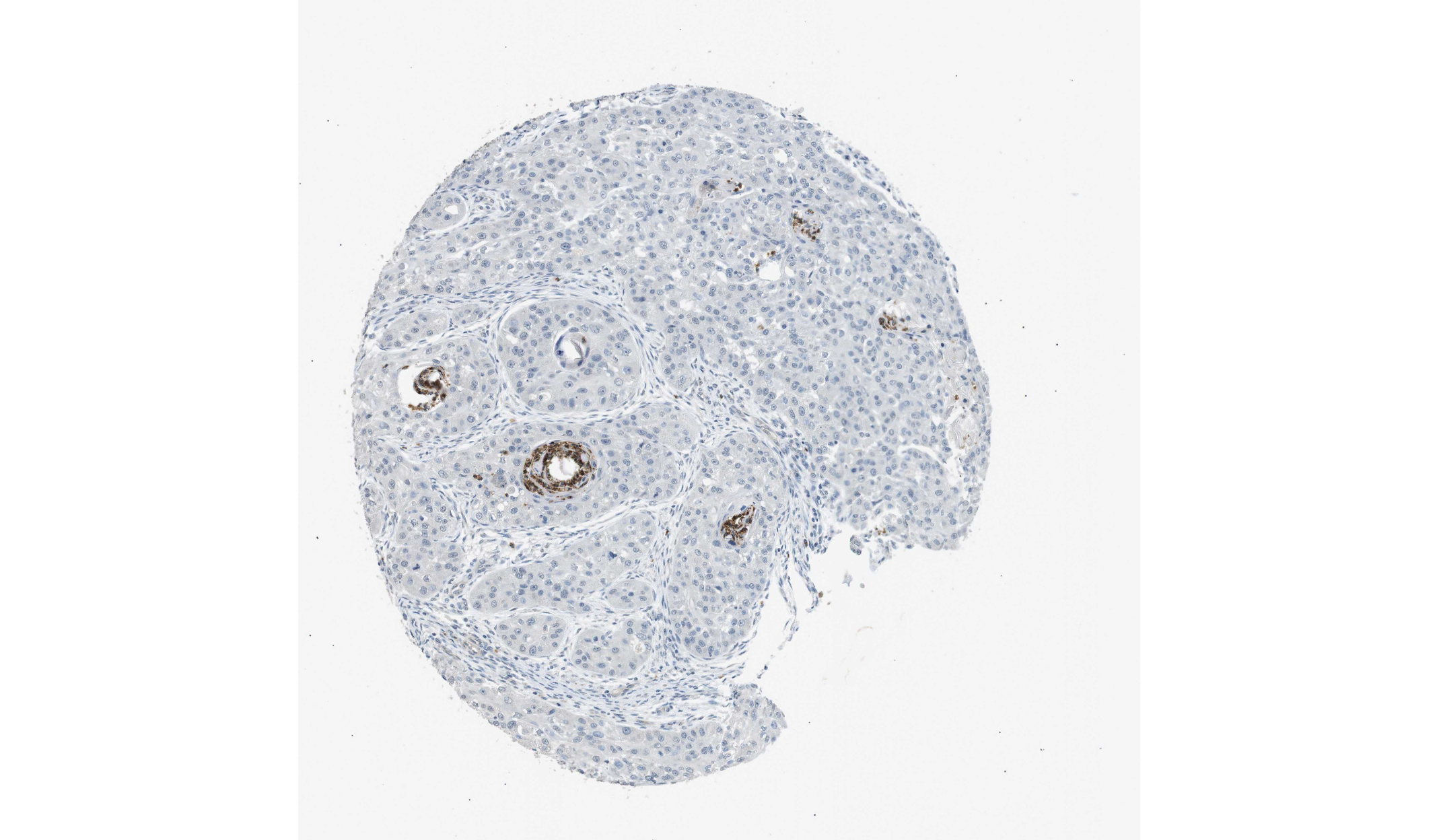

Supplement: Supplementary file 2 [file DataSheet4.ZIP › Immunohistochemistry(1)/GZMMTumor.png]

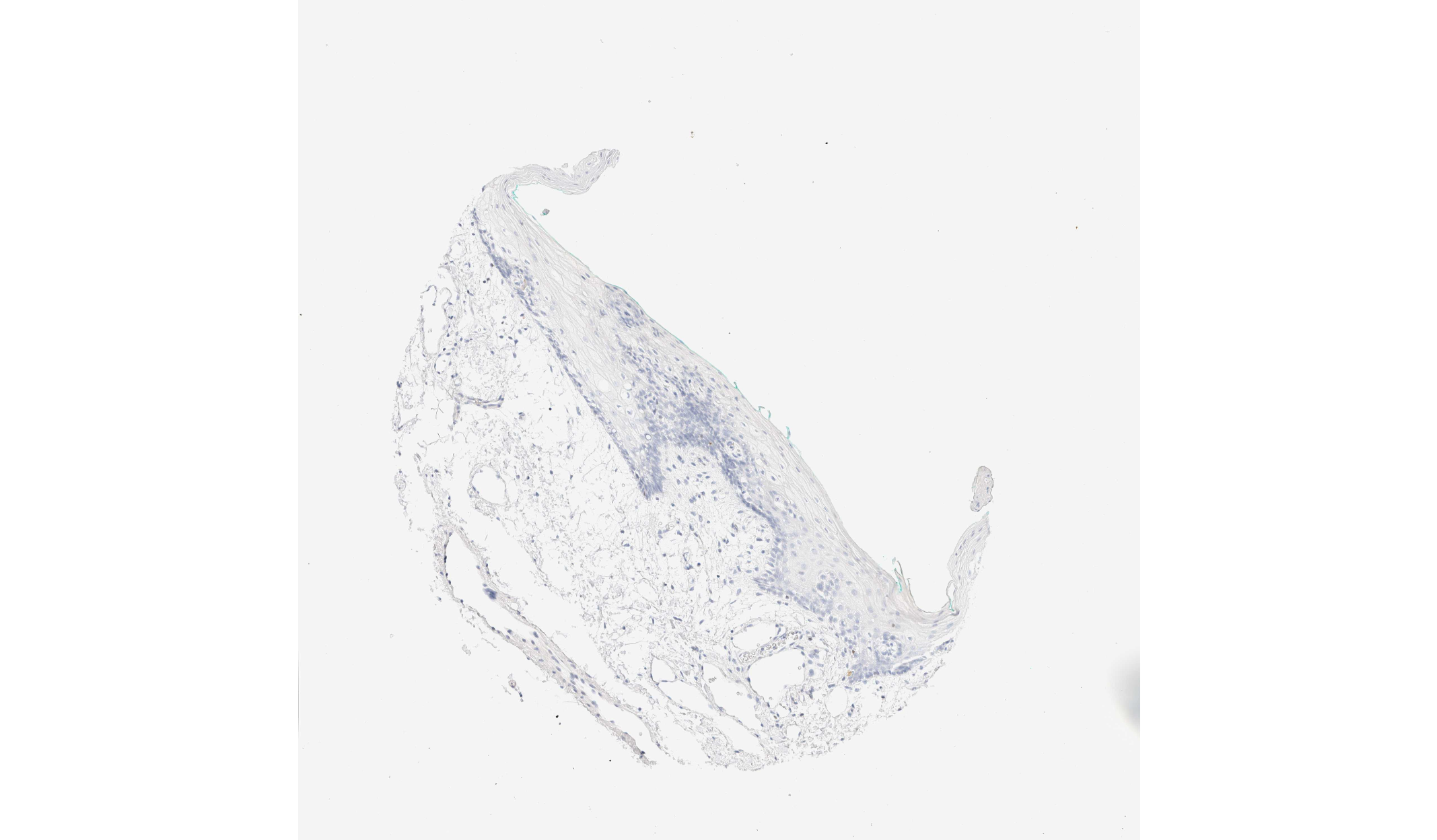

Supplement: Supplementary file 5 [file DataSheet5.ZIP › Immunohistochemistry(2)/IKZF3Normal.png]

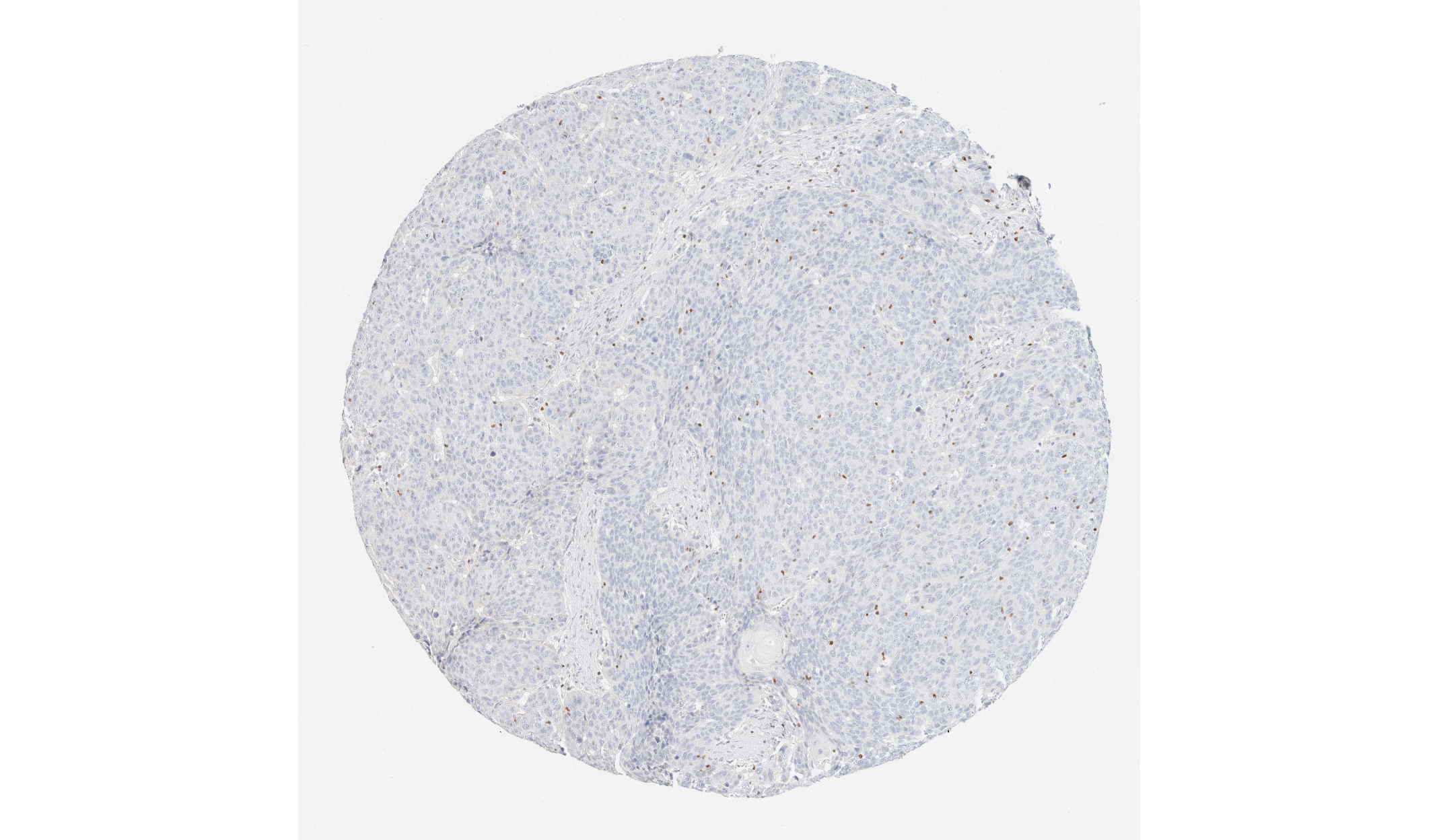

Supplement: Supplementary file 5 [file DataSheet5.ZIP › Immunohistochemistry(2)/IKZF3Tumor.png]

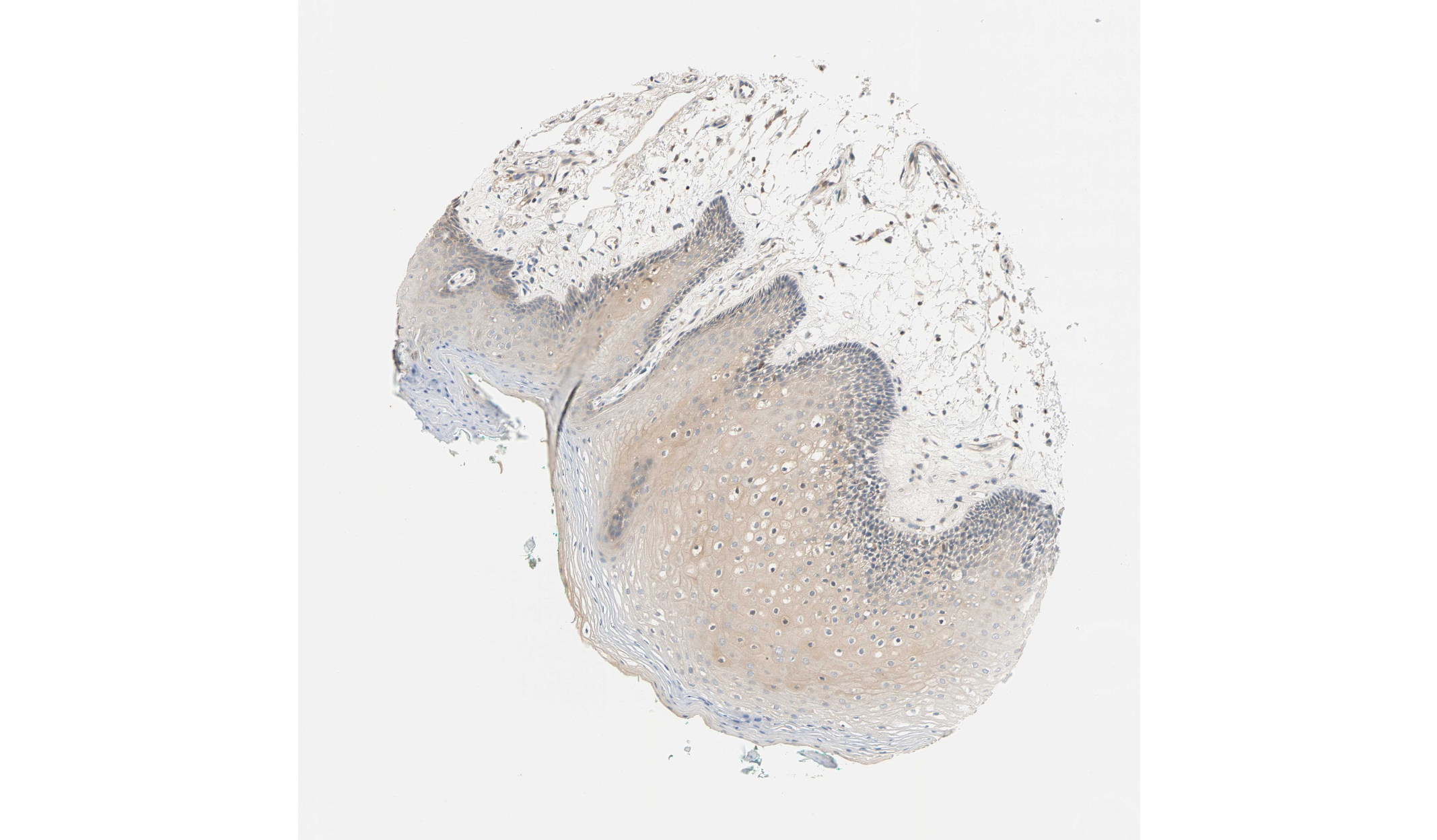

Supplement: Supplementary file 5 [file DataSheet5.ZIP › Immunohistochemistry(2)/MAP4K1Normal.png]

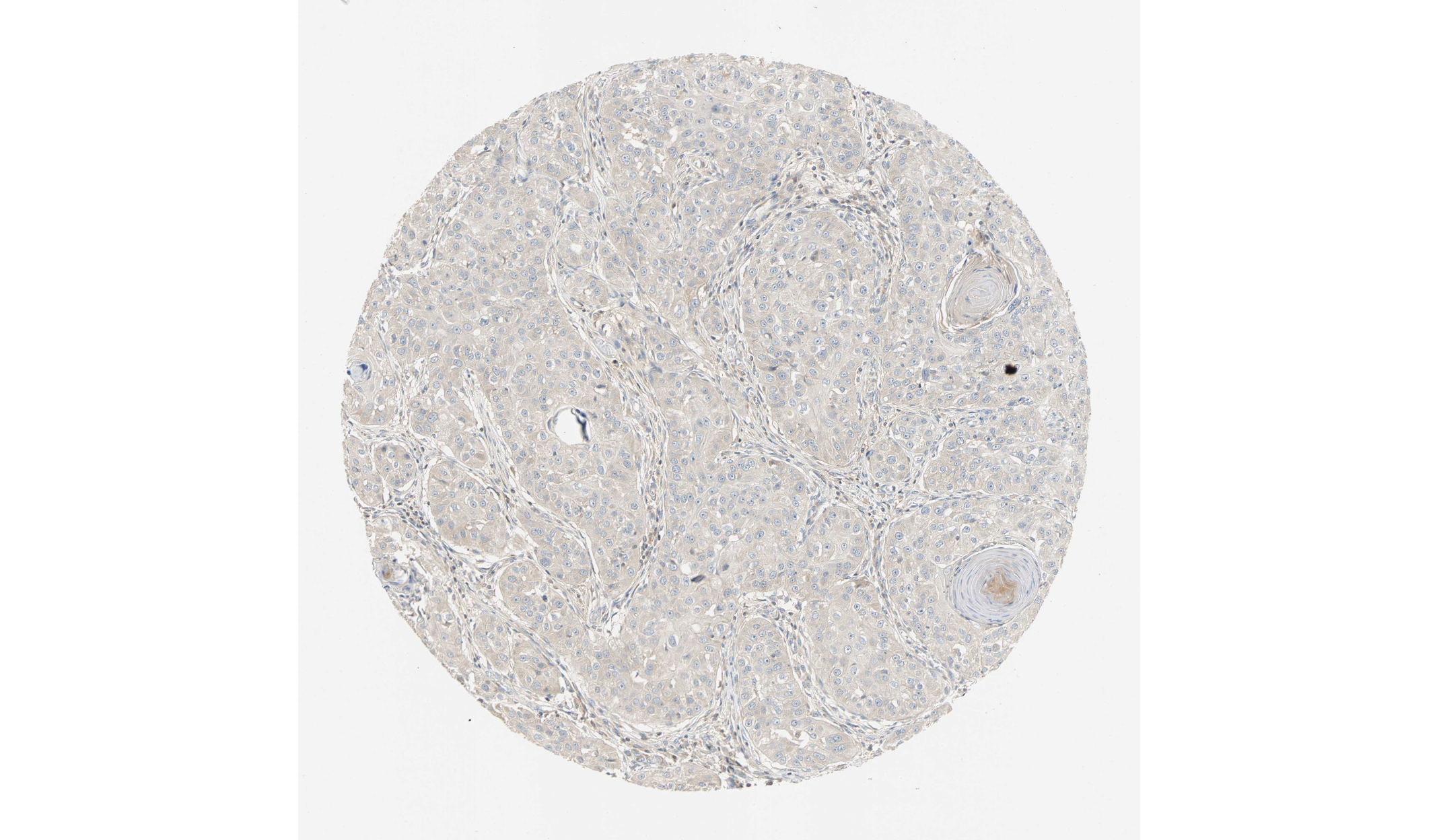

Supplement: Supplementary file 5 [file DataSheet5.ZIP › Immunohistochemistry(2)/MAP4K1Tumor.png]

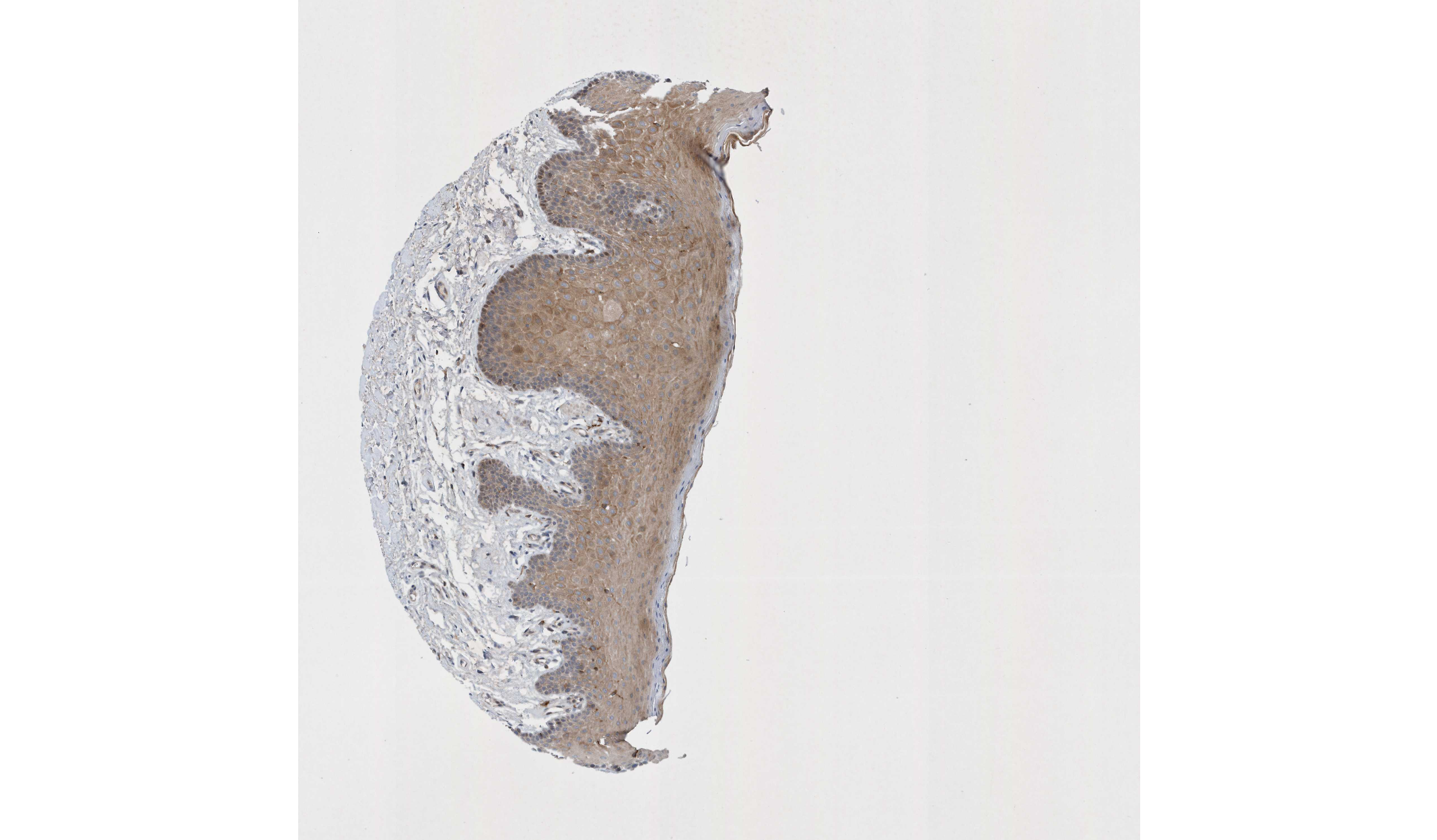

Supplement: Supplementary file 5 [file DataSheet5.ZIP › Immunohistochemistry(2)/P2RY10Normal.png]

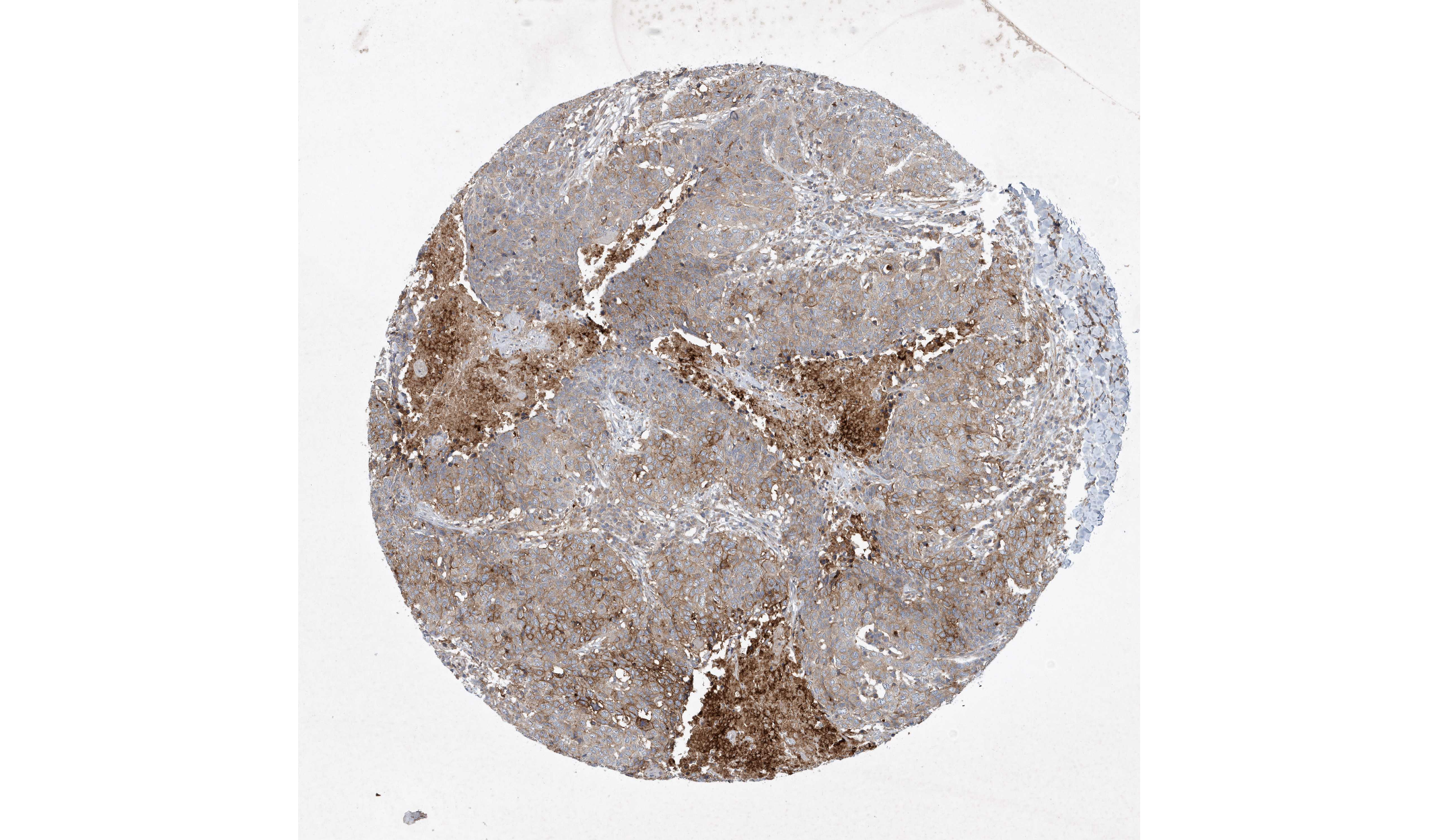

Supplement: Supplementary file 5 [file DataSheet5.ZIP › Immunohistochemistry(2)/P2RY10Tumor.png]

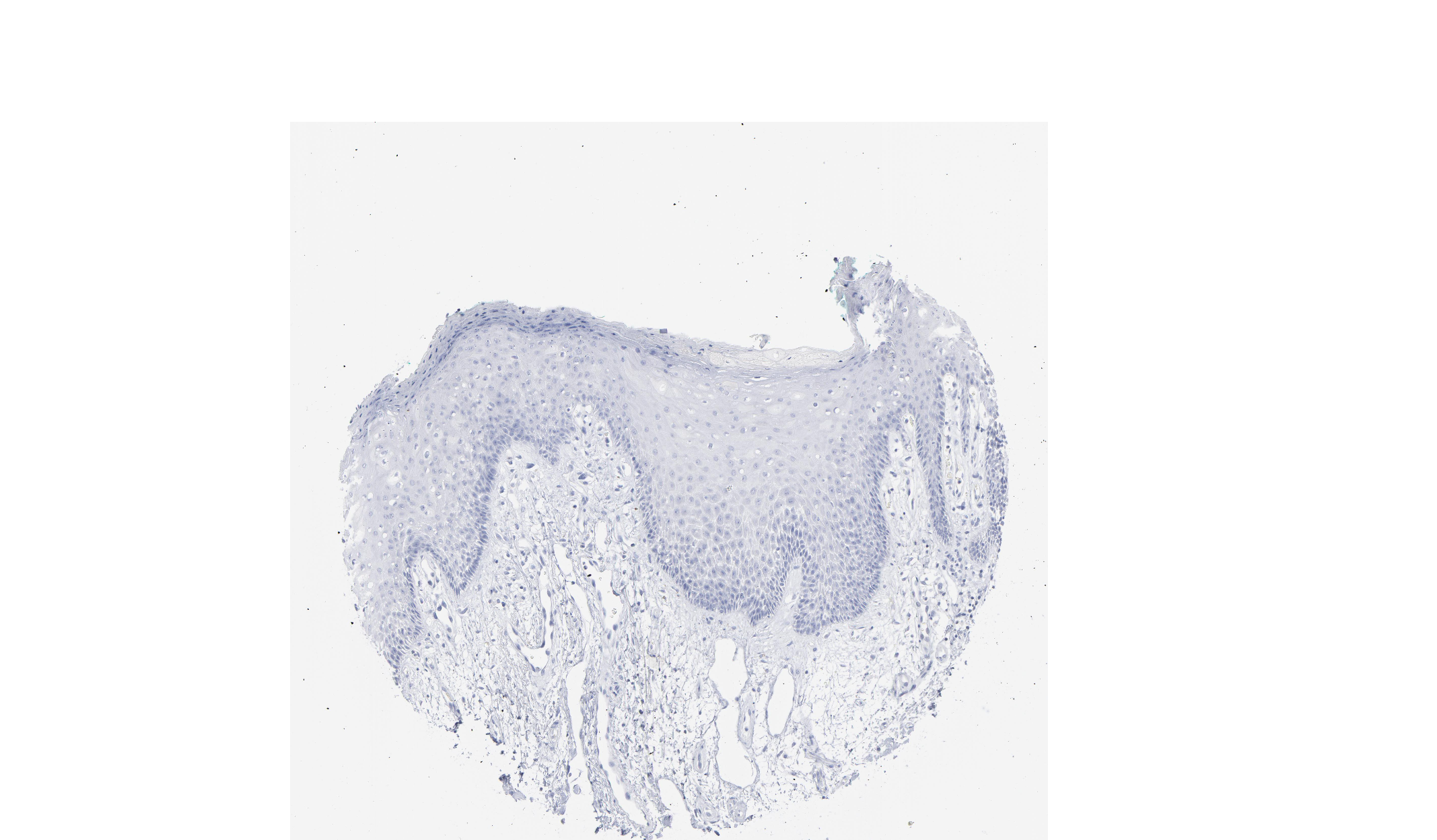

Supplement: Supplementary file 5 [file DataSheet5.ZIP › Immunohistochemistry(2)/P2RY8Normal.png]

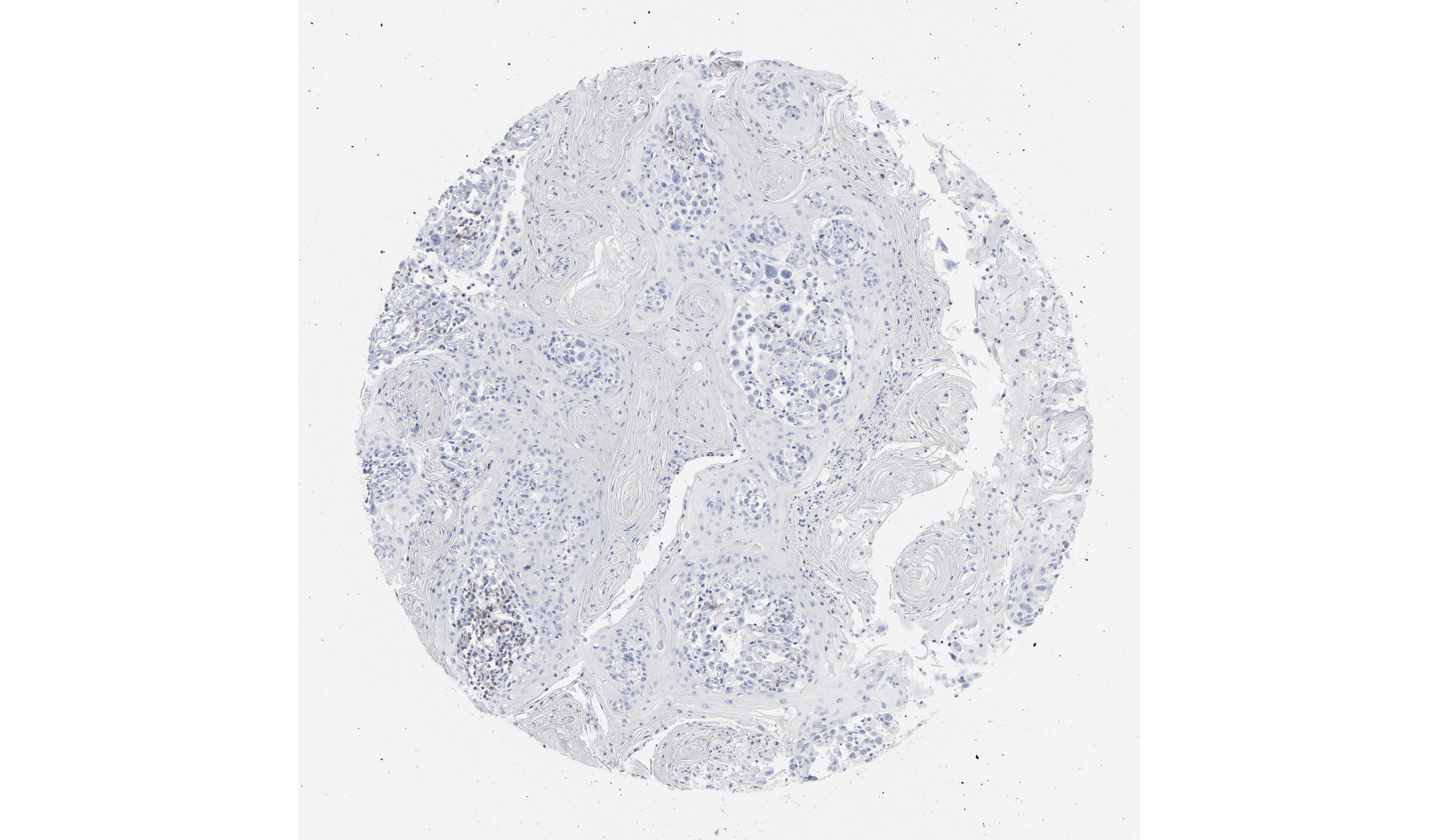

Supplement: Supplementary file 5 [file DataSheet5.ZIP › Immunohistochemistry(2)/P2RY8Tumor.png]

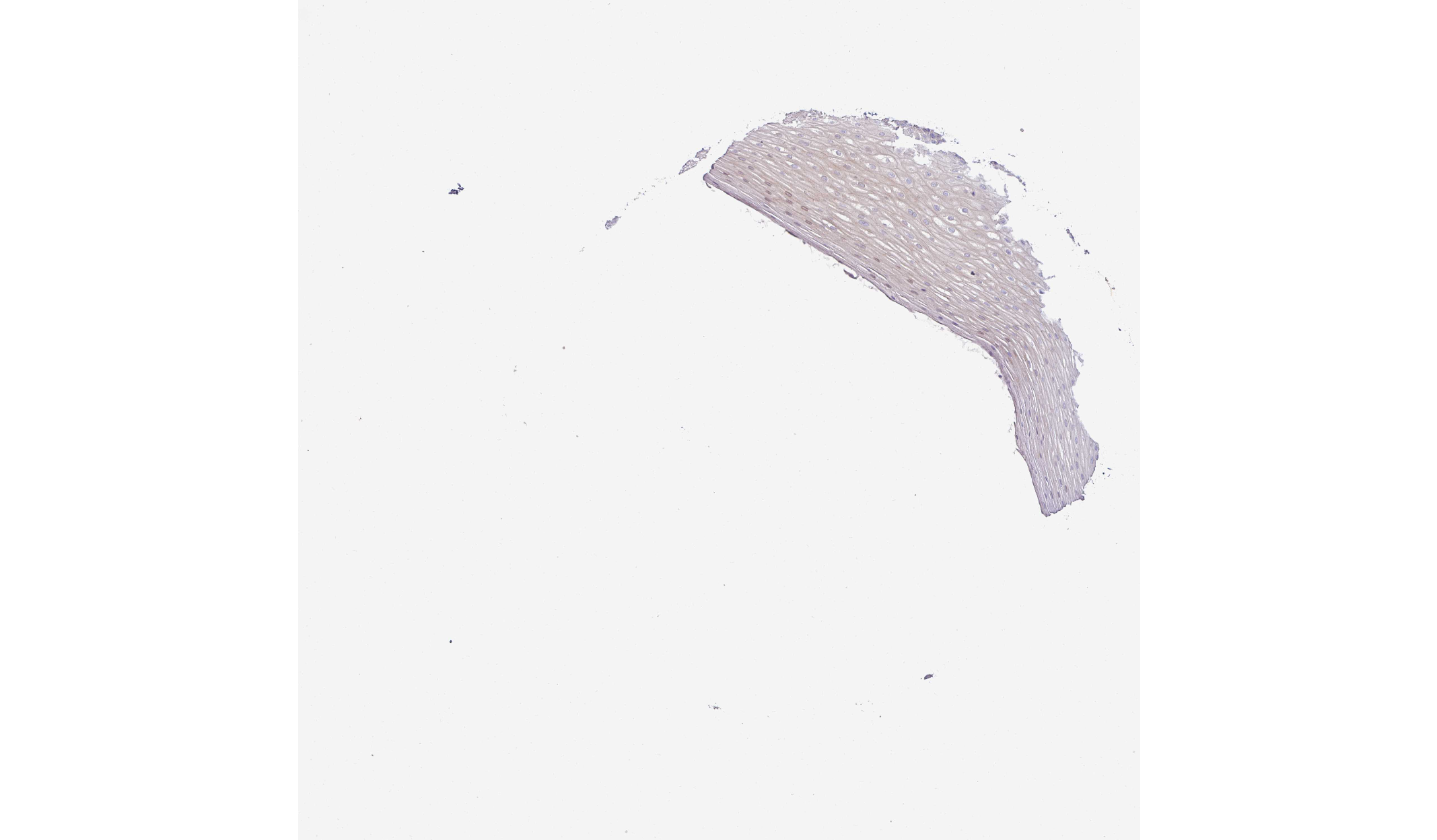

Supplement: Supplementary file 5 [file DataSheet5.ZIP › Immunohistochemistry(2)/SLAMF6Normal.png]

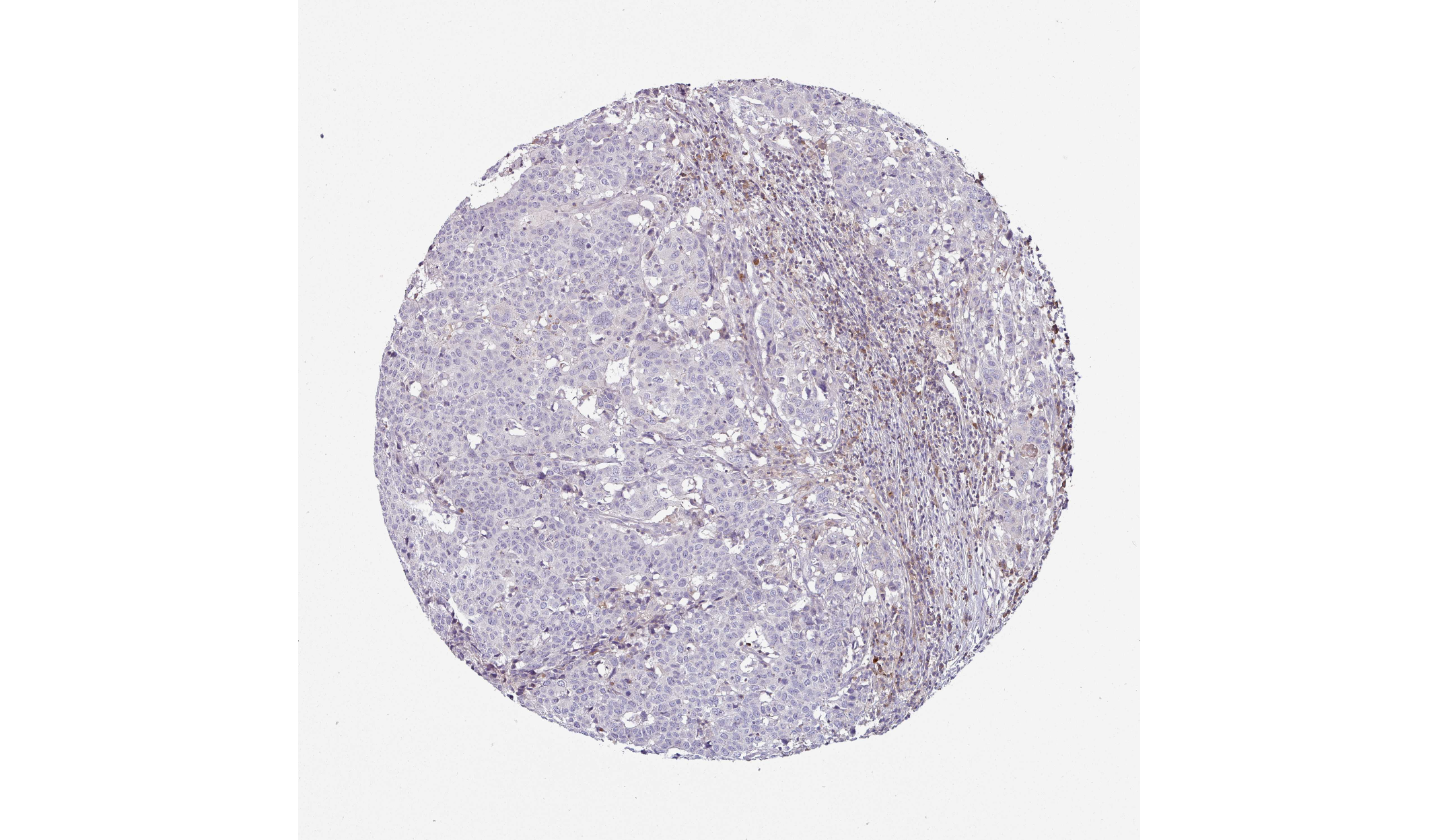

Supplement: Supplementary file 5 [file DataSheet5.ZIP › Immunohistochemistry(2)/SLAMF6Tumor.png]

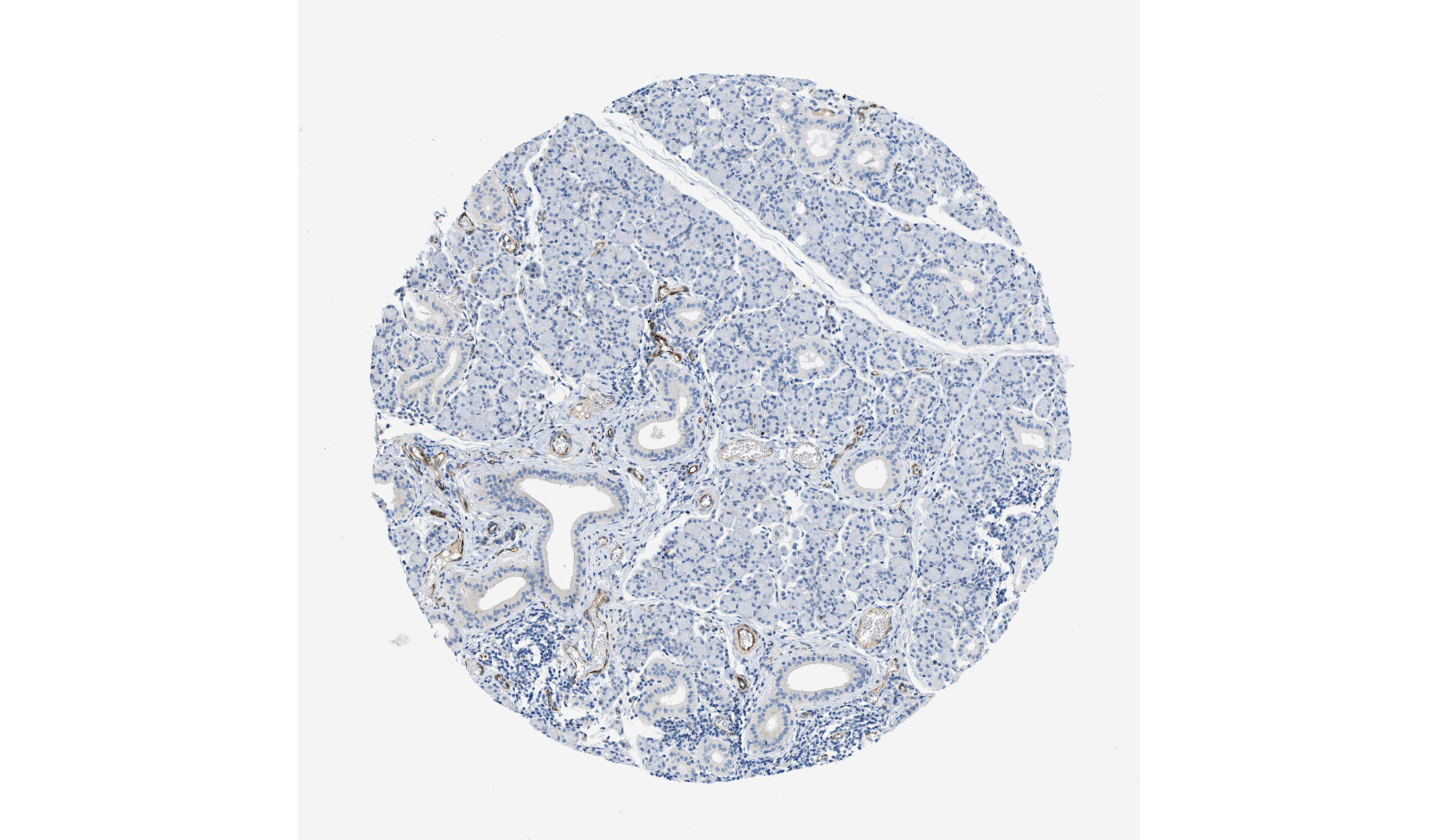

Supplement: Supplementary file 5 [file DataSheet5.ZIP › Immunohistochemistry(2)/XCR1Normal.png]

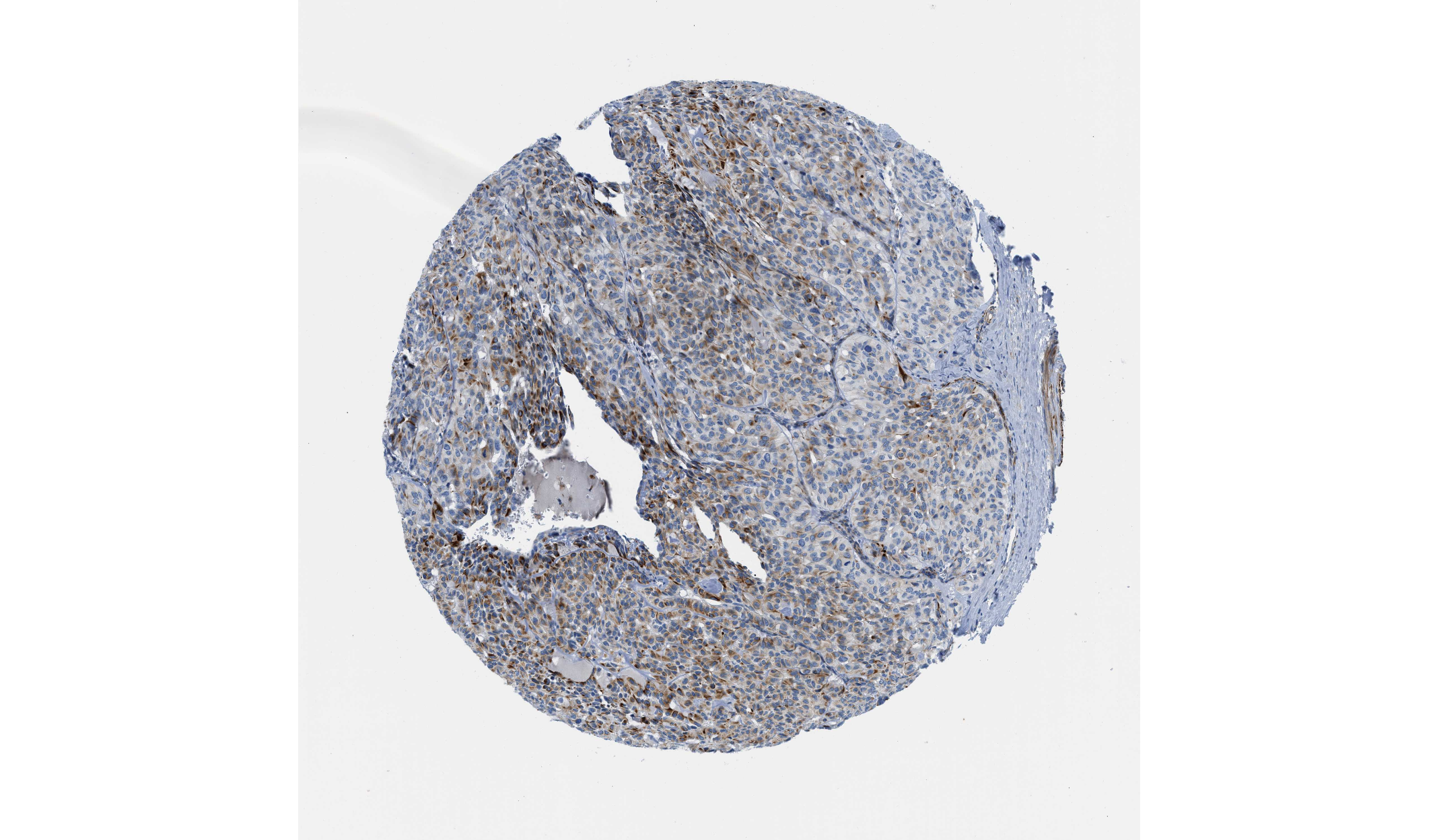

Supplement: Supplementary file 5 [file DataSheet5.ZIP › Immunohistochemistry(2)/XCR1Tumor.png]
